# Efficacy and Safety of Treatments for Different Stages of Syphilis: a Systematic Review and Network Meta-Analysis of Randomized Controlled Trials and Observational Studies

**DOI:** 10.1128/spectrum.02977-22

**Published:** 2022-11-15

**Authors:** Meixiao Liu, Yuxin Fan, Jingjing Chen, Jiaru Yang, Li Gao, Xinya Wu, Xin Xu, Yu Zhang, Peng Yue, Wenjing Cao, Zhenhua Ji, Xuan Su, Shiyuan Wen, Jing Kong, Guozhong Zhou, Bingxue Li, Yan Dong, Aihua Liu, Fukai Bao

**Affiliations:** a The Institute for Tropical Medicine, Faculty of Basic Medical Science, Kunming Medical University, Kunming, China; b Yunnan Province Key Laboratory of Children's Major Diseases Research, The Affiliated Children Hospital, Kunming Medical University, Kunming, China; Taichung Veterans General Hospital

**Keywords:** syphilis, antibiotics, treatment, efficacy, safety, network meta-analysis

## Abstract

Parenteral penicillin is the first-line regimen for treating syphilis. However, allergic reactions and poor drug tolerance still present challenging problems with respect to use of this antibiotic. This study aimed to evaluate the efficacy and safety of ceftriaxone, erythromycin, minocycline, tetracycline, and doxycycline for syphilis treatment, compared with penicillin, to determine which antibiotic could be a better substitute for penicillin. This study included 17 articles, comprising 3 randomized controlled trials (RCTs) and 14 observational studies and involving 4,485 syphilis patients. Estimated risk ratios (RRs) and 95% confidence interval (CIs) were used to compare the serological response rates. At the 6- and 12-month follow-ups, the serological response rates were compared by direct meta-analysis and network meta-analysis (NMA). Based on direct meta-analysis, the serological response rates at the 3- and 24-month follow-ups were compared. Our NMA showed a higher serological response rate for ceftriaxone than for penicillin at the 6-month follow-up (RR of 1.12, 95% CI of 1.02 to 1.23). Ceftriaxone was equally effective as penicillin for syphilis in terms of serological response rates, and it was a better substitute for penicillin than ceftriaxone, erythromycin, minocycline, tetracycline, or doxycycline. However, more large-scale, high-quality, double-blind trials are still needed to determine whether ceftriaxone can safely replace penicillin for the treatment of syphilis when necessary.

**IMPORTANCE** Parenteral penicillin is the first-line regimen for syphilis treatment. However, allergic reactions and poor drug tolerance still present emerging threatening problems with respect to use of this antibiotic. Our results showed a higher serological response rate for ceftriaxone than for penicillin at the 6-month follow-up. Sufficient data are not available for demonstrating significant differences in the efficacy of the other four antibiotics (erythromycin, minocycline, tetracycline, and doxycycline) for treating syphilis. In the clinical treatment of syphilis in patients who are allergic to penicillin or for whom penicillin is not available, ceftriaxone appears to be a better alternative treatment. This meta-analysis provides a reference for clinical treatment of syphilis. Currently, a lack of sufficient evidence to guide antibiotic treatment of syphilis exists, and a need for more high-quality RCTs is still present. This network meta-analysis can lay a foundation for further research.

## INTRODUCTION

Syphilis is a chronic, systemic sexually transmitted disease caused by Treponema pallidum subsp*. pallidum.* Syphilis is prevalent worldwide. According to an estimation by the World Health Organization (WHO), about 12 million new cases occur every year in the world (1). T. pallidum can invade a variety of tissues and organs and remain there for a long time, and it can induce a variety of clinical manifestations, which include primary, secondary, tertiary, latent, and congenital forms of syphilis. T. pallidum can also be transmitted to the fetus through the placenta of a pregnant woman, causing an intrauterine infection of the fetus that leads to abortion, premature birth, stillbirth, or delivery of a child with congenital syphilis. Notably, syphilis has been shown to help increase the risk of acquiring and transmitting human immunodeficiency virus (HIV) infection (1–4). Syphilis is therefore a key issue for global public health.

At present, the treatment of syphilis mainly depends on antibiotics. Several penicillins, including procaine and benzathine penicillins, are the preferred drugs for treating syphilis that is in different stages (5). However, allergic reactions, poor drug tolerance (6, 7), and limited patient efficacy remain challenging issues (8). As antibiotic resistance increases (9) and the number of newer antibiotics available decreases, the pressure to choose suitable antibiotics is greater in patients with a history of adverse reactions. Therefore, it is of vital importance to find alternative antibiotics that can perform as well as or outperform penicillins.

Five alternative antibiotics (ceftriaxone, erythromycin, minocycline, tetracycline, and doxycycline) were selected for the treatment of syphilis based on literature searches and the 2020 European Guideline on the Management of Syphilis (10). The purpose of this study was to determine which antibiotic is a better alternative to penicillin by evaluating the efficacy and safety of five alternative antibiotics (ceftriaxone, erythromycin, minocycline, tetracycline, and doxycycline) relative to that of penicillin in the treatment of syphilis based on a network meta-analysis (NMA).

## RESULTS

### Study selection.

**FIG 1:**
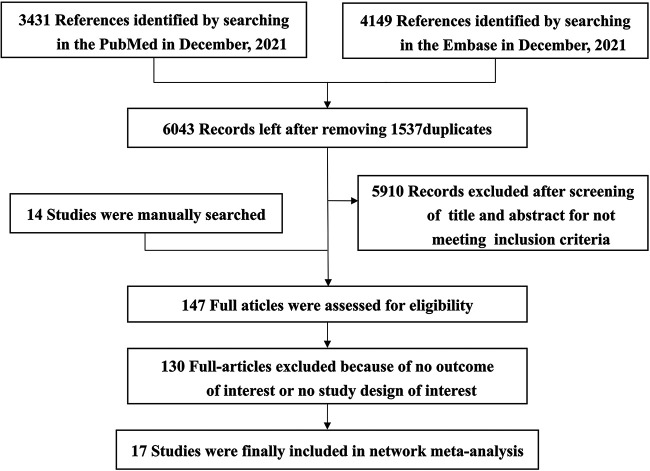
Flow diagram for the referred reporting system for systemic reviews and meta-analysis (PRISMA).

Pubmed and Embase databases were searched, and 3,431 and 4,149 articles, respectively, were retrieved. After excluding 1,537 duplicates, the titles and abstracts of 6,043 studies were screened. With the addition of 14 manually retrieved studies, a total of 147 studies were read in full, resulting in the inclusion of 17 eligible reports involving 4,485 syphilis patients (Fig. 1).

### Study characteristics.

A total of 4,485 patients of ages between 14 and 80 years and primary, secondary, or delayed syphilis were enrolled in this study. Of the 4,485 patients, 3,083 received intramuscular or oral penicillin, 323 received ceftriaxone, 286 received erythromycin, 314 received minocycline, 257 received tetracycline, and 222 received doxycycline (Table 1).

**TABLE 1:** Characteristics of the included studies

Reference	Study type	Stage or type of syphilis	Interventions	No. of patients	Dosage and duration^*a*^
Bettuzzi 2021 (14)	Retrospective cohort	Neurosyphilis	Penicillin	166	BenPen, 3–4 MU i.v. every 4 h
			Ceftriaxone	42	2 g i.v. once daily for 10 days
Wu 2021 (20)	Retrospective cohort	Primary, secondary, or early latent	Penicillin	118	BenPen, 2.4 MU i.m. once a wk, 1–2 times
			Minocycline	158	Oral, 100 mg twice daily for 28 days
Antonio 2019 (21)	Retrospective cohort	Early, late latent, or latent	Penicillin	115	BenPen, 240,000 IU i.m. or 240,000 IU wkly, 3 wks
			Doxycycline	50	Oral, 100 mg twice daily for 14 or 28 days
Cao 2017 (11)	RCT	Primary, secondary, or early latent	Penicillin	151	BenPen, 2.4 MU i.m. wkly for 2 wks
			Ceftriaxone	150	1 g i.v. once daily for 10 days
Shao 2016 (17)	Retrospective cohort	Primary, secondary, or early latent	Penicillin	40	BenPen, 2.4 MU i.m. single dose
			Minocycline	156	Oral, 100 mg twice daily for 14 or 28 days
Tsai 2014 (16)	Retrospective cohort	Primary, secondary, or early latent	Penicillin	271	BenPen, 2.4 MU i.m. once
			Doxycycline	123	Oral, 100 mg twice daily for 14 days
Psomas 2012 (19)	Retrospective cohort	Primary, secondary, or early latent	Penicillin	52	BenPen, 2.4 MIU i.m. once per wk for 1, 2, or 3 wks
			Doxycycline	15	Oral, 100 mg 2 or 3 times/day, for 14 to 21 days
			Ceftriaxone	49	1 or 2 g i.v. once/day for 14 to 21 days
Spornraft-Ragaller 2011 (22)	Retrospective cohort	Primary, secondary, or early latent	Penicillin	12	BenPen, 2.4 MU i.m. once/wk for 2 or 3 wks, 1 MU i.m. daily for 14–21 days, or 10 MU i.v. 3× daily for 21 days
			Ceftriaxone	12	1–2 g/day i.v. for 10–21 days
Ghanem 2006 (18)	Retrospective cohort	Primary, secondary, or early latent	Penicillin	73	BenPen, 2.4 MU i.m. single dose
			Doxycycline	34	Oral, 200 mg/day for 14 days
Dowell 1992 (23)	Retrospective cohort	Primary, secondary, or early latent, neurosyphilis	Penicillin	13	BenPen, 2.4 MU once/wk for 3 wks
			Ceftriaxone	43	1 to 2 g/day for 10–14 days
Schofer 1989 (12)	RCT	Primary or secondary	Penicillin	14	BenPen, 1 MIU i.m. daily for 15 days
			Ceftriaxone	14	4 × 1 g i.m. every 2 days
Moorthy 1987 (13)	RCT	Primary	Penicillin	5	BenPen, 2.4 MU, single i.m.
			Ceftriaxone	13	3 g (single i.m.), 2 g/day i.m. for 2 days, or 2 g/day i.m. for 5 days
Fiumara 1978 (25)	Retrospective cohort	Early latent	Penicillin	252	BenPen, 2.4 MU i.m. wkly for 2 wks
			Tetracycline	23	Oral, 2 g/day for 12 days
Fiumara 1977 (26)	Retrospective cohort	Secondary	Penicillin	165	BenPen, 2.4 MU i.m. wkly for 2 wks
			Tetracycline	39	Oral, 2 g/day for 12 days
Fiumara 1977 (27)	Retrospective cohort	Primary	Penicillin	175	BenPen, 2.4 MU i.m. wkly for 2 wks
			Tetracycline	21	Oral, 2 g/day for 12 days
Schroeter 1972 (24)	Prospective cohort	Primary or secondary	Penicillin	262	BenPen, 2.4 MU i.m. (single dose), procaine penicillin G with aluminum stearate in total 4.8 MU, or aqueous procaine penicillin G in total 4.8 MU
			Tetracycline	107	Oral, total of 30 g (3 g/day for 10 days)
			Erythromycin	215	Oral, total of 20 g (2 g/day for 10 days)
Lucas 1967 (15)	Retrospective cohort	Primary or secondary	Penicillin	178	BenPen 2.4 MU i.m., procaine penicillin G in oil with aluminum monostearate 4.8 MU total, or aqueous procaine penicillin G 4.8 MU total
			Erythromycin	71	Oral, 20 g over 10–15 days
			Tetracycline	67	Oral, 30 g over 10–15 days

a BenPen, benzathine penicillin G; i.m., intramuscular injection; i.v., intravenous injection; MU, million units.

### Quality evaluation of included research.

A total of 17 articles were included in the study: three randomized controlled trials (RCTs) (11–13) and 14 observational studies (14–27). Randomization and open labeling were mentioned for all three RCTs included (see Fig. S1 and Fig. S2 in the supplemental material). Sixteen observational studies that met most of the quality evaluation criteria were included. These 16 cohort studies had Newcastle-Ottawa scale (NOS) quality scores ranging from 6 to 9, with a mean score of 7.3 ± 0.7 (± standard deviation). Among them, the NOS quality score of one article was 9, for two articles the score was 8, for 10 articles it was 7, and one article was scored a 6. Therefore, most of the cohort study literature was of a high quality level. Five studies used statistical methods to control for significant confounding factors during the analysis. Nine studies were followed for up to 2 years (Table S4).

### Head-to-head meta-analysis of serological response rates.

**(i) Serological response rate at 3-month follow-up.** Six trials reported serological response data at the 3-month follow-up. Direct meta-analysis results showed a similar serological response rate for the four interventions (ceftriaxone versus penicillin: RR 1.05, 95% CI 0.92 to 1.20; tetracycline versus penicillin: RR 1.01, 95% CI 0.99 to 1.04; erythromycin versus penicillin: RR 0.98, 95% CI 0.95 to 1.00) as shown in Tables S5 and S6.

**(ii) Serological response rate at 6-month follow-up.** Eleven trials reported serological response data at the 6-month follow-up. With the exception of ceftriaxone, the other five interventions had comparable serological response rates (tetracycline versus penicillin: RR 0.98, 95% CI 0.93 to 1.03; doxycycline versus penicillin: RR 0.88, 95% CI 0.75 to 1.02; erythromycin versus penicillin: RR 0.91, 95% CI 0.80 to 1.03). Ceftriaxone demonstrated a better serological response rate than penicillin (ceftriaxone versus penicillin: RR 1.13, 95% CI 1.03 to 1.25) as shown in Table S5 and S6 in the supplemental material.

**FIG 2:**
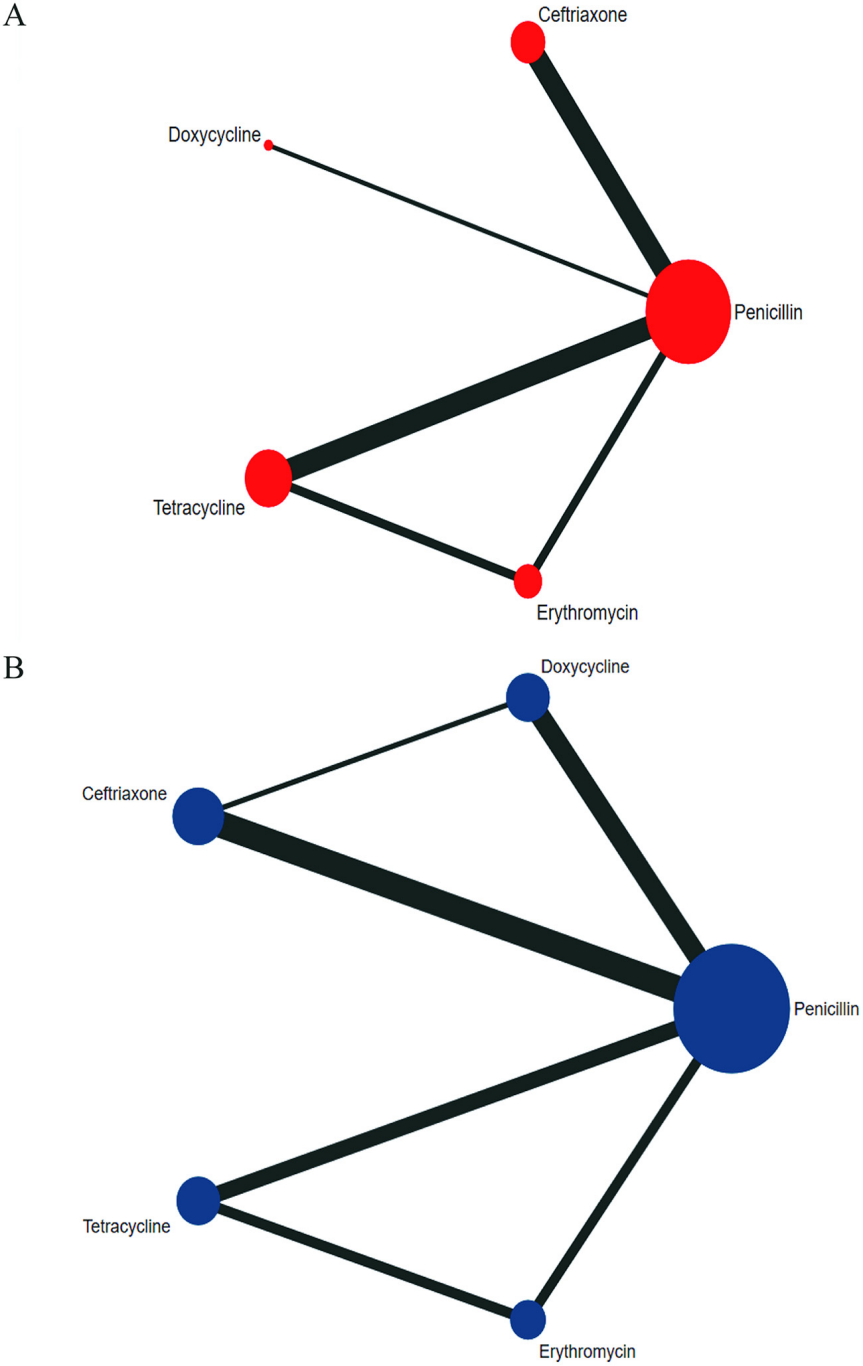
Network diagram of serological response rates for 6-month follow-up (A) and 12-month follow-up (B).

**FIG 3:**
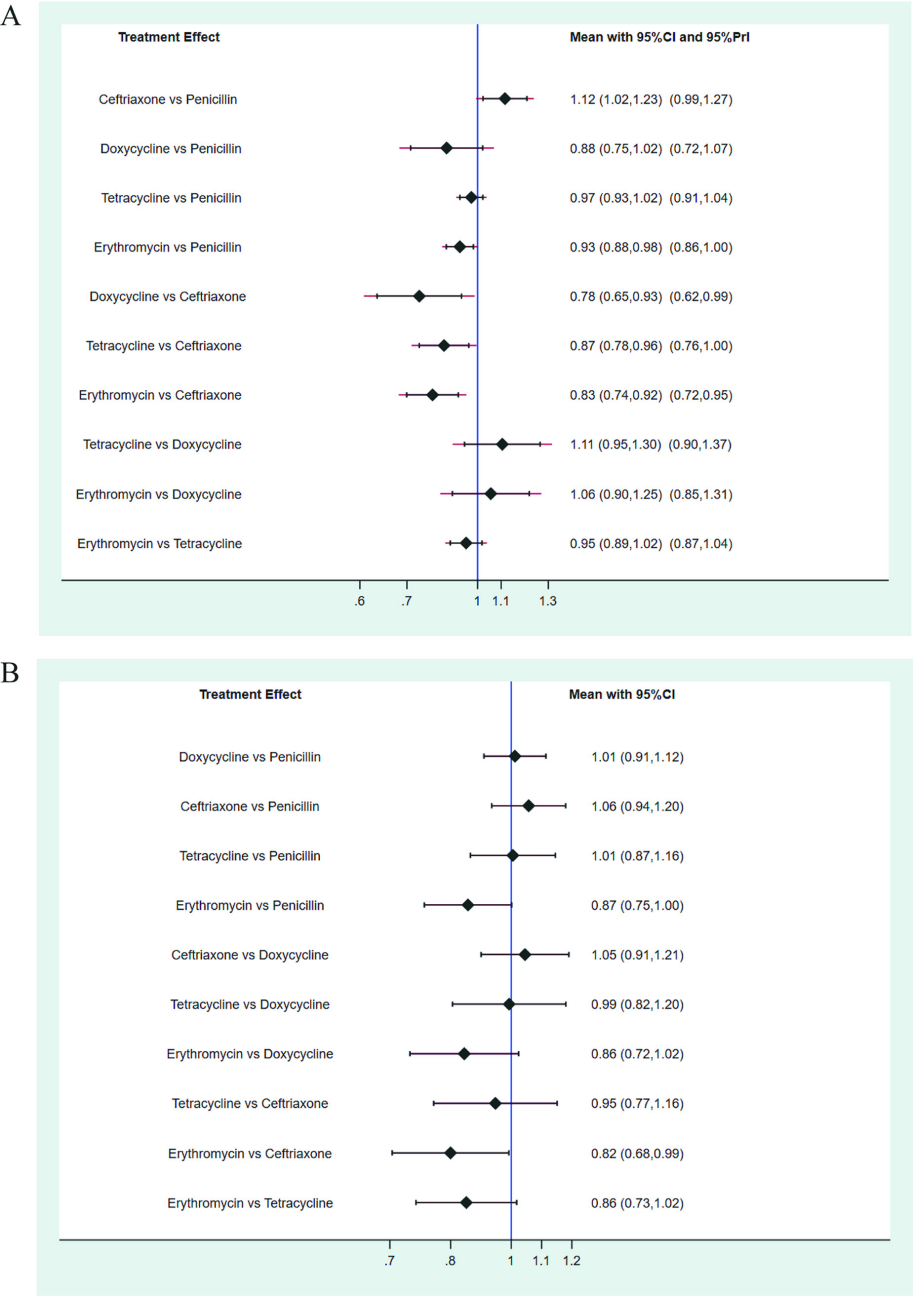
Summary of network meta-analyses of serological response rates at 6-month follow-up (A) and 12-month follow-up (B).

Figure 2 illustrates the network of five antibiotics involved in the 6-month follow-up analysis. The NMA results showed that the serological response rate in the ceftriaxone group was higher than in the penicillin group (RR 1.12, 95% CI 1.02 to 1.23) at the 6-month follow-up. The efficacy comparisons of doxycycline versus penicillin (RR 0.88, 95% CI 0.75 to 1.02), tetracycline versus penicillin (RR 0.97, 95% CI 0.93 to 1.02), and erythromycin versus penicillin (RR 0.93, 95% CI 0.88 to 0.98) are shown in Fig. 3. The inconsistency test based on the node-splitting method (*P* > 0.05) and loop inconsistency showed that the results of direct comparisons were consistent with those of the indirect comparison, and no statistical inconsistency was found (Table S7). The ranking results of the five intervention measures were in the following descending order: ceftriaxone > penicillin > tetracycline > erythromycin > doxycycline (see Tables S8 and Fig. S3 in the supplement material). The funnel plot results showed that most of the research scatter points were located above the funnel plot and indicated a biased distribution, suggesting that the above results have a certain publication bias. At the same time, some scatter points could be found at the bottom of the funnel plot, indicating that the above results may have been affected by the small-sample effect (Fig. S4).

A subgroup analysis of ceftriaxone versus penicillin for neurosyphilis and early syphilis, to further understand the efficacy of ceftriaxone and penicillin for neurosyphilis and early syphilis treatment, was performed. The treatment options for neurosyphilis are shown in Table S9. Five studies reported a comparison of penicillin and ceftriaxone for syphilis at the 6-month follow-up. Both direct meta-analysis and NMA indicated that ceftriaxone had better efficacy than penicillin. Subgroup analysis showed that the serological response rate to ceftriaxone in the treatment of neurosyphilis was better than that of penicillin (RR 1.07, 95% CI 0.90 to 1.26), but not statistically significant. There was no statistical significance in the treatment of early syphilis (RR 1.00, 95% CI 0.65 to 1.53) (Fig. S5).

**(iii) Serological response rate at 12-month follow-up.** Twelve trials reported serological response data at the 12-month follow-up. Direct meta-analysis results showed similar serological response rates for the five interventions (ceftriaxone versus penicillin: RR 1.03, 95% CI 0.91 to 1.17; tetracycline versus penicillin: RR 1.04, 95% CI 0.87 to 1.25; doxycycline versus penicillin: RR 1.04, 95% CI 0.98 to 1.10; erythromycin versus penicillin: RR 0.64, 95% CI 0.28 to 1.46) as shown in Tables S5 and S6.

Figure 2 shows the construction of a network of five antibiotics involved at the 12-month follow-up. NMA results showed no significant differences in serological response rates between any two of the five treatments at the 12-month follow-up. The efficacies of doxycycline and penicillin (RR 1.01, 95% CI 0.91 to 1.12), ceftriaxone and penicillin (RR 1.06, 95% CI 0.94 to 1.20), tetracycline and penicillin (RR 1.01, 95% CI 0.87 to 1.16), and erythromycin and penicillin (RR 0.87, 95% CI 0.75 to 1.00) are shown in Fig. 3. The inconsistency test by node-splitting method (*P* > 0.05) and loop inconsistency showed that the results of direct comparison were consistent with those of indirect comparison, and no statistical inconsistency existed (Table S7). The ranking results of the five intervention antibiotics were in the following descending order: ceftriaxone > doxycycline > tetracycline > penicillin > erythromycin (Table S8 and Fig. S3). The funnel plot results showed that most of the research scatter points were located above the funnel plot and revealed a biased distribution, suggesting that the above results had certain publication bias. At the same time, some scatter points were located at the bottom of the funnel plot, indicating that the above results may have been affected by a small-sample effect (Fig. S4).

**(iv) Serological response rate at 24-month follow-up.** Five trials reported serological response data at the 24-month follow-up. Direct meta-analysis results yielded a similar serological response rate for the four interventions (minocycline versus penicillin: RR 0.99, 95% CI 0.90 to 1.07; tetracycline versus penicillin: RR 1.00, 95% CI 0.96 to 1.03; erythromycin versus penicillin: RR 0.83, 95% CI 0.67 to 1.02), as shown in Tables S5 and S6.

### Qualitative analysis for drug side effects of syphilis treatment.

Only 3 of the 17 included studies reported drug safety. Due to the lack of reported data, only qualitative analyses were conducted in our study. These three studies reported adverse reactions caused by penicillin and ceftriaxone during the treatment of syphilis with a sample size of 359 people, and the main adverse reactions were the Jarisch-Herzheimer reaction and penicillin allergy. Two of these studies showed a higher incidence of Jarisch-Herzheimer reactions after ceftriaxone treatment, rather than penicillin, for syphilis (Table 2).

**TABLE 2:** Overview of adverse drug reactions^*a*^

Study	Interventions	Route of administration	Adverse events	No. of incidents	Sample size	Incidence
Schofer 1989 (12)	Penicillin	i.m., 1 MIU daily, for 15 days	Allergic penicillin exanthema	1	14	7%
	Ceftriaxone	i.m., 4 × 1 g, every 2 days		0	14	0%
Psomas 2012 (19)	Penicillin	i.m. 2.4 MIU, daily at 1-wk interval	Jarisch-Herzheimer reaction	0	52	0%
	Ceftriaxone	i.v., 1–2 g, 2–3 times daily, for 14–21 days		3	49	6%
Cao 2017 (11)	Penicillin	i.m., 2.4 MU, wkly for 2 wks	Jarisch-Herzheimer reaction	37	118	31%
	Ceftriaxone	i.v., 1 g daily for 10 days		46	112	41%

a i.m., intramuscular injection; i.v., intravenous injection.

## DISCUSSION

Although benzathine penicillin is still recognized as the first-line treatment for syphilis, its high patient allergy rate and episodic shortage prevents some patients from receiving such treatment (10). For a long time, the search for alternative treatments for syphilis has attracted the attention of many researchers.

The current study was based on 3 RCTs and 14 retrospective studies, comprising 4,485 patients with syphilis assigned to treatment with six individual antibiotics. Because the 2020 European Guideline on the Management of Syphilis excluded azithromycin as an alternative treatment for any stage of syphilis, the ceftriaxone, erythromycin, minocycline, tetracycline, and doxycycline options were chosen as alternative treatment target drugs (10). Direct meta-analyses and NMA of serological response rates to antibiotic treatment of syphilis according to different follow-up periods were performed. The NMA showed that ceftriaxone treatment produced a slightly better serological response rate than penicillin at 6 months, and the surface under the cumulative ranking curve (SUCRA) results suggested that ceftriaxone may be the antibiotic with the highest serological response rate for treating syphilis. These response rates were in the following descending order: ceftriaxone > penicillin > tetracycline > erythromycin > doxycycline. This may have been because ceftriaxone is a third-generation broad-spectrum high-efficiency cephalosporin with a rapid effect and a long half-life after entering the organism and has a strong killing effect on syphilis spirochetes (28, 29). However, no statistical differences in serological response among the five antibiotics at the 12-month follow-up were noted. This finding was consistent with the results of the direct meta-analysis.

In addition, the results of our subgroup analysis showed that the serological response rate to ceftriaxone for neurosyphilis treatment was superior to that with penicillin, but the difference was not statistically significant. Compared with other antibiotics, ceftriaxone has higher lipid solubility and higher penetration into tissues and extracellular membranes (30). These characteristics enable it to quickly cross the blood-brain barrier into the cerebrospinal fluid, and its effective drug concentration is maintained for a longer time to kill Treponema pallidum. The European guidelines for the treatment of syphilis recommend ceftriaxone as the second-line agent for the treatment of neurosyphilis (10). When penicillin is unavailable or patients are allergic to it, WHO recommends treatment with ceftriaxone, doxycycline, or azithromycin (31). In addition, in the treatment of congenital syphilis, azithromycin cannot cross the placenta, so the fetus is not treated with azithromycin, and doxycycline cannot be used in pregnant women because of its adverse effects (31). Therefore, ceftriaxone is considered by WHO to be the optimal option and can be injected for pregnant women with Treponema pallidum infection. However, the 2020 European Guideline on the Management of Syphilis, which was written after the WHO guideline, excluded azithromycin as an alternative treatment for any stage of syphilis (10).

A direct meta-analysis of serological response rates after treatment at 3 months of follow-up was also conducted and showed similar efficacies of all five drugs. This suggested that several antibiotics are equally effective in early infections, but more evidence is needed. The treatment safety analysis was based on the incidence of adverse events after treatment. The results showed that the incidence of Jarisch-Herzheimer reaction after ceftriaxone treatment was higher than after penicillin administration. Of all the studies we included, only two involved Jarisch-Herzheimer reactions. Given the limited data available, the Jarisch-Herzheimer reactions may be related to the route and the interval of drug administration. Intramuscular injections and short intervals seem more likely to induce the Jarisch-Herzheimer reactions. More clinical studies are still needed to assess the safety of antibiotics to treat patients with syphilis.

A previous study by Liu et al. used NMA to evaluate the effectiveness of three antibiotics (penicillin, ceftriaxone, and doxycycline-tetracycline) for syphilis treatment. The results showed no significant differences in the efficacies of the three antibiotics (29). These results differed somewhat from ours, probably because the study by Liu et al. only included nine papers involving 2,049 syphilis patients, with sample sizes that were relatively small; furthermore, their study was limited to early syphilis. Compared with previous literature studies, we included more articles with larger sample sizes and more drugs analyzed, which allowed us to obtain more evidence. To our knowledge, our study is the first time that the safety of 5 antibiotics in the treatment of syphilis has been evaluated. Furthermore, a subgroup analysis for different syphilis forms was performed. However, due to the small sample size and insufficient data, it should be stressed that more studies are still needed to validate these and future results.

Some limitations can be found in our study. First, most of the included studies were observational, indicating that results were more likely to be influenced by confounding factors than the results from the RCTs. However, a modified NOS was used to assess the quality of each study, and the included observational studies were of medium to high quality. High heterogeneity was also not observed in our results. Second, although more studies were included in our study, the number was still relatively small, which may have reduced the reliability of the results, especially for the safety of treatment.

Overall, this study showed that ceftriaxone was more effective than penicillin in treating syphilis after 6 months, but its safety profile was lower. When penicillin was not available, ceftriaxone was an effective treatment alternative for syphilis. Sufficient data demonstrating the significant differences in the efficacies of the other four antibiotics for syphilis are still not available, and more clinical studies are needed to support their use as alternatives to penicillin.

## MATERIALS AND METHODS

### Ethics statement.

This research was performed according to the Preferred Reporting Items for Systematic Reviews and Meta-Analyses (PRISMA) guidelines.

### Retrieval strategy.

We combined the medical topic title (MeSH) descriptor with free-text terms to conduct a systematic computerized literature search on RCTs and observational studies involving syphilis patients treated with penicillin, ceftriaxone, erythromycin, minocycline, tetracycline, or doxycycline, and we made appropriate adjustments according to changes in the database. References from the meta-analyses of included articles and records were retrieved to expand the scope of our literature search (see Tables S1, S2, and S3 in the supplemental material).

### Inclusion criteria.

Eligible articles included in this study must have fulfilled several criteria: (i) the articles were published RCTs or observational research in English; (ii) they involved primary, secondary, tertiary, or latent syphilis; and (iii) they compared penicillin to the alternative antibiotics (ceftriaxone, erythromycin, minocycline, tetracycline, or doxycycline).

### Data extraction.

Two investigators independently completed study selection, data extraction, and quality assessment for inclusion in the study. Any differences were resolved through discussion. The resulting article titles and abstracts were reviewed, eligible articles according to inclusion criteria were selected, and their full texts were then read. Several pieces of information were extracted from the included studies using Excel tables: (i) first author, (ii) year of publication, (iii) study type, (iv) syphilis stage, (v) basic information for the patient, (vi) medication information (mode and dose of medication), (vii) prognosis and efficacy, and (viii) adverse reactions.

Of interest were the serological response rates to syphilis treatment at follow-up intervals of 3, 6, 12, and 24 months and the safety of different antibiotic treatments. Serological response was defined as conversion of titer to negative or a ≥4-fold (two dilutions) reduction in the venereal disease research laboratory test/rapid plasma recovery test (VDRL/RPR) during the follow-up period.

### Quality assessment.

The quality of identified RCTs was evaluated using the Cochrane risk assessment tool for bias (32). Risk map for bias were generated using Review Manager 5.4 (Cochrane Collaboration, Oxford, United Kingdom). A modified Newcastle-Ottawa scale (NOS) to assess the quality of each cohort study was applied. The modified NOS consists of three sections and nine items: (i) selection (0 to 4 points), (ii) comparability (0 to 2 points), and (iii) outcome evaluation (0 to 3 points). Studies with NOS scores between 1 and 3 were considered low quality, those with scores between 4 and 6 were considered medium quality, and those with scores between 7 and 9 were considered high quality.

### Statistical methods.

Statistical analyses were performed using Review Manager 5.4 (Cochrane Collaboration), STATA 17.0 (College Station, TX, USA), and the meta package in R (version 4.1.3). Network meta-analysis is a generalization of pairwise meta-analysis that can be used to compare all pairs of treatments within a number of treatments for the same condition (33, 34). We evaluated the efficacy and safety of antibiotics for syphilis by using an NMA (35, 36). All extracted data were analyzed using random effect models and consistency models. Relative risk and 95% confidence interval were indicators of efficacy and safety, respectively, in this NMA study. Inconsistency was another important metric that needed to be assessed by the NMA. Estimates of the effects of direct and indirect evidence to assess NMA inconsistencies were compared. If the *P* value was <0.05, the NMA was inconsistent. This NMA inconsistency was examined by evaluating the differences in estimates between direct and indirect evidence using the node-splitting method (36, 37). Cumulative probability ranking of antibiotic efficacy was performed to measure the degree of certainty regarding the superiority of one treatment over the other (38). This study was registered with PROSPERO, number CRD42022302549.
