# Supplementary material for: Efficacy and Safety of Treatments for Different Stages of Syphilis: a Systematic Review and Network Meta-Analysis of Randomized Controlled Trials and Observational Studies
Source: Microbiol Spectr. 2022 Nov 15;10(6):e02977-22. doi: 10.1128/spectrum.02977-22 (PMC9769634; doi:10.1128/spectrum.02977-22)
Supplement: Supplemental file 1 — Supplemental material. Download spectrum.02977-22-s0001.pdf, PDF file, 0.7 MB [file spectrum.02977-22-s0001.pdf]

Supplemental Material for Efficacy and safety of treatments for different stages of syphilis: A systematic review and network meta-analysis of randomized controlled trials and observational studies

Content list

eTable 1 PRISMA2020 checklist

eTable 2 PubMed search strategy and result

eTable 3 Embase search strategy and result

eTable 4 Results of quality assessment using Newcastle

eTable 5 Direct meta-analysis for different follow-up time

eTable 6 Serological response rate data of intervention measures in different follow-up time

eTable 7 Loop inconsistency test results

eTable 8 Serological response rates league table at the 6-month follow-up and 12-month follow-up

eTable 9 Treatment options for neurosyphilis

eFigure 1 Risk of bias summary

eFigure 2 Risk of bias graph

eFigure 3 Cumulative ranking probability curves of serological response rates at 6-month follow-up (A) and 12-month follow-up (B)

eFigure 4 Funnel plots of serological response rates at the 6-month follow-up (A) and 12-month follow-up (B)

eFigure 5 Subgroup analysis of ceftriaxone versus penicillin for treatment of neurosyphilis and early syphilis at the 6-month follow-up

Section and Topic	Item #	Checklist item	Location where item is reported
TITLE			
Title	1	Identify the report as a systematic review.	1
ABSTRACT			
Abstract	2	See the PRISMA 2020 for Abstracts checklist.	2
INTRODUCTION			
Rationale	3	Describe the rationale for the review in the context of existing knowledge.	3
Objectives	4	Provide an explicit statement of the objective(s) or question(s) the review addresses.	4
METHODS			
Eligibility criteria	5	Specify the inclusion and exclusion criteria for the review and how studies were grouped for the syntheses.	5
Information sources	6	Specify all databases, registers, websites, organisations, reference lists and other sources searched or consulted to identify studies. Specify the date when each source was last searched or consulted.	5,12
Search strategy	7	Present the full search strategies for all databases, registers and websites, including any filters and limits used.	5
Selection process	8	Specify the methods used to decide whether a study met the inclusion criteria of the review, including how many reviewers screened each record and each report retrieved, whether they worked independently, and if applicable, details of automation tools used in the process.	5
Data collection process	9	Specify the methods used to collect data from reports, including how many reviewers collected data from each report, whether they worked independently, any processes for obtaining or confirming data from study investigators, and if applicable, details of automation tools used in the process.	5
Data items	10a	List and define all outcomes for which data were sought. Specify whether all results that were compatible with each outcome domain in each study were sought (e.g. for all measures, time points, analyses), and if not, the methods used to decide which results to collect.	5
	10b	List and define all other variables for which data were sought (e.g. participant and intervention characteristics, funding sources). Describe any assumptions made about any missing or unclear information.	5
Study risk of	11	Specify the methods used to assess risk of bias in the included studies,	5,6

bias assessment		including details of the tool(s) used, how many reviewers assessed each study and whether they worked independently, and if applicable, details of automation tools used in the process.	
Effect measures	12	Specify for each outcome the effect measure(s) (e.g. risk ratio, mean difference) used in the synthesis or presentation of results.	6
Synthesis methods	13a	Describe the processes used to decide which studies were eligible for each synthesis (e.g. tabulating the study intervention characteristics and comparing against the planned groups for each synthesis (item #5)).	7
	13b	Describe any methods required to prepare the data for presentation or synthesis, such as handling of missing summary statistics, or data conversions.	6
	13c	Describe any methods used to tabulate or visually display results of individual studies and syntheses.	6
	13d	Describe any methods used to synthesize results and provide a rationale for the choice(s). If meta-analysis was performed, describe the model(s), method(s) to identify the presence and extent of statistical heterogeneity, and software package(s) used.	6
	13e	Describe any methods used to explore possible causes of heterogeneity among study results (e.g. subgroup analysis, meta-regression).	6
	13f	Describe any sensitivity analyses conducted to assess robustness of the synthesized results.	Not applicable
Reporting bias assessment	14	Describe any methods used to assess risk of bias due to missing results in a synthesis (arising from reporting biases).	6
Certainty assessment	15	Describe any methods used to assess certainty (or confidence) in the body of evidence for an outcome.	6
RESULTS			
Study selection	16a	Describe the results of the search and selection process, from the number of records identified in the search to the number of studies included in the review, ideally using a flow diagram.	6,17
	16b	Cite studies that might appear to meet the inclusion criteria, but which were excluded, and explain why they were excluded.	6
Study characteristics	17	Cite each included study and present its characteristics.	7
Risk of bias in studies	18	Present assessments of risk of bias for each included study.	7
Results of individual	19	For all outcomes, present, for each study: (a) summary statistics for each group (where appropriate) and (b) an effect estimate and its precision (e.g.	15,16

studies		confidence/credible interval), ideally using structured tables or plots.	
Results of syntheses	20a	For each synthesis, briefly summarise the characteristics and risk of bias among contributing studies.	7
	20b	Present results of all statistical syntheses conducted. If meta-analysis was done, present for each the summary estimate and its precision (e.g. confidence/credible interval) and measures of statistical heterogeneity. If comparing groups, describe the direction of the effect.	7-10
	20c	Present results of all investigations of possible causes of heterogeneity among study results.	Not applicable
	20d	Present results of all sensitivity analyses conducted to assess the robustness of the synthesized results.	Not applicable
Reporting biases	21	Present assessments of risk of bias due to missing results (arising from reporting biases) for each synthesis assessed.	Not applicable
Certainty of evidence	22	Present assessments of certainty (or confidence) in the body of evidence for each outcome assessed.	7-10
DISCUSSION			
Discussion	23a	Provide a general interpretation of the results in the context of other evidence.	10
	23b	Discuss any limitations of the evidence included in the review.	11
	23c	Discuss any limitations of the review processes used.	11
	23d	Discuss implications of the results for practice, policy, and future research.	11,12
OTHER INFORMATION			
Registration and protocol	24a	Provide registration information for the review, including register name and registration number, or state that the review was not registered.	12
	24b	Indicate where the review protocol can be accessed, or state that a protocol was not prepared.	12
	24c	Describe and explain any amendments to information provided at registration or in the protocol.	Not applicable
Support	25	Describe sources of financial or non-financial support for the review, and the role of the funders or sponsors in the review.	12
Competing interests	26	Declare any competing interests of review authors.	12
Availability of data, code and other materials	27	Report which of the following are publicly available and where they can be found: template data collection forms; data extracted from included studies; data used for all analyses; analytic code; any other materials used in the review.	

From: Page MJ, McKenzie JE, Bossuyt PM, Boutron I, Hoffmann TC, Mulrow CD, et al. The PRISMA 2020 statement: an updated guideline for reporting systematic reviews. BMJ 2021;372:n71. doi: 10.1136/bmj.n71

For more information, visit: http://www.prisma-statement.org/

eTable 1 PRISMA2020 checklist

Search number	Query	Results	Time
1	"Syphilis"[Mesh]	28,705	3:38:09
2	Great Pox[Title/Abstract]	11	3:38:29
3	("Syphilis"[Mesh]) OR (Great Pox[Title/Abstract])	28,707	3:38:52
4	"Penicillins"[Mesh]	82,673	3:39:46
5	((Antibiotics, Penicillin[Title/Abstract]) OR (Penicillin Antibiotics[Title/Abstract])) OR (Penicillin[Title/Abstract])	56,571	3:40:29
6	("Penicillins"[Mesh]) OR (((Antibiotics, Penicillin[Title/Abstract]) OR (Penicillin Antibiotics[Title/Abstract])) OR (Penicillin[Title/Abstract]))	106,776	3:41:17
7	"Ceftriaxone"[Mesh]	6,445	3:42:00
8	((((((((((((((((((((((((((((Ceftriaxon[Title/Abstract]) OR (Cefatriaxone[Title/Abstract])) OR (Ceftriaxone, Disodium Salt, Hemiheptahydrate[Title/Abstract])) OR (Ceftrex[Title/Abstract])) OR (Ceftriaxon Curamed[Title/Abstract])) OR (Ceftriaxon Hexal[Title/Abstract])) OR (Ceftriaxona Andreu[Title/Abstract])) OR (Ceftriaxona LDP Torlan[Title/Abstract])) OR (Ceftriaxone Irex[Title/Abstract])) OR (Ceftriaxone Sodium[Title/Abstract])) OR (Ceftriaxone Sodium, Anhydrous[Title/Abstract])) OR (Anhydrous Ceftriaxone Sodium[Title/Abstract])) OR (Lendacin[Title/Abstract])) OR (Longacef[Title/Abstract])) OR (Longaceph[Title/Abstract])) OR (Ro13-9904[Title/Abstract])) OR (Ro13 9904[Title/Abstract])) OR (Ro139904[Title/Abstract])) OR (Ro-13-9904[Title/Abstract])) OR (Ro 13-9904[Title/Abstract])) OR (Ro 13 9904[Title/Abstract])) OR (Ro 139904[Title/Abstract])) OR (Rocephin[Title/Abstract])) OR (Rocefalin[Title/Abstract])) OR (Rocephine[Title/Abstract])) OR (Rocefin[Title/Abstract])) OR (Tacex[Title/Abstract])) OR (Terbac[Title/Abstract])) OR (Ceftriaxone, Disodium Salt[Title/Abstract])) OR (Benaxona[Title/Abstract])) OR (Cefaxona[Title/Abstract])	548	3:47:55
9	("Ceftriaxone"[Mesh]) OR ((((((((((((((((((((((((((((Ceftriaxon[Title/Abstract]) OR (Cefatriaxone[Title/Abstract])) OR (Ceftriaxone, Disodium Salt, Hemiheptahydrate[Title/Abstract])) OR (Ceftrex[Title/Abstract])) OR (Ceftriaxon Curamed[Title/Abstract])) OR (Ceftriaxon Hexal[Title/Abstract])) OR (Ceftriaxona Andreu[Title/Abstract])) OR (Ceftriaxona LDP	6,688	3:48:23

	Torlan[Title/Abstract])) OR (Ceftriaxone Irex[Title/Abstract])) OR (Ceftriaxone Sodium[Title/Abstract])) OR (Ceftriaxone Sodium, Anhydrous[Title/Abstract])) OR (Anhydrous Ceftriaxone Sodium[Title/Abstract])) OR (Lendacin[Title/Abstract])) OR (Longacef[Title/Abstract])) OR (Longaceph[Title/Abstract])) OR (Ro13-9904[Title/Abstract])) OR (Ro13 9904[Title/Abstract])) OR (Ro139904[Title/Abstract])) OR (Ro-13-9904[Title/Abstract])) OR (Ro 13-9904[Title/Abstract])) OR (Ro 13 9904[Title/Abstract])) OR (Ro 139904[Title/Abstract])) OR (Rocephin[Title/Abstract])) OR (Rocefalin[Title/Abstract])) OR (Rocephine[Title/Abstract])) OR (Rocefin[Title/Abstract])) OR (Tacex[Title/Abstract])) OR (Terbac[Title/Abstract])) OR (Ceftriaxone, Disodium Salt[Title/Abstract])) OR (Benaxona[Title/Abstract])) OR (Cefaxona[Title/Abstract]))		
10	"Erythromycin"[Mesh]	26,662	3:55:01
11	((((((((((Erythromycin A[Title/Abstract]) OR (Erythromycin Phosphate[Title/Abstract])) OR (Phosphate, Erythromycin[Title/Abstract])) OR (Erythromycin Lactate[Title/Abstract])) OR (Lactate, Erythromycin[Title/Abstract])) OR (T-Stat[Title/Abstract])) OR (T Stat[Title/Abstract])) OR (TStat[Title/Abstract])) OR (Erymax[Title/Abstract])) OR (Erythromycin C[Title/Abstract])) OR (Erycette[Title/Abstract])) OR (Ilotycin[Title/Abstract])	987	3:57:48
12	("Erythromycin"[Mesh]) OR (((((((((((Erythromycin A[Title/Abstract]) OR (Erythromycin Phosphate[Title/Abstract])) OR (Phosphate, Erythromycin[Title/Abstract])) OR (Erythromycin Lactate[Title/Abstract])) OR (Lactate, Erythromycin[Title/Abstract])) OR (T-Stat[Title/Abstract])) OR (T Stat[Title/Abstract])) OR (TStat[Title/Abstract])) OR (Erymax[Title/Abstract])) OR (Erythromycin C[Title/Abstract])) OR (Erycette[Title/Abstract])) OR (Ilotycin[Title/Abstract]))	27,008	3:58:38
13	"Minocycline"[Mesh]	6,217	3:59:33
14	(((((((((((((((((((((((((((((((((((Minox 50[Title/Abstract]) OR (Aknemin[Title/Abstract])) OR (Aknin-Mino[Title/Abstract])) OR (Aknin Mino[Title/Abstract])) OR (Aknosan[Title/Abstract])) OR (Mynocine[Title/Abstract])) OR (Apo-Minocycline[Title/Abstract])) OR (Apo Minocycline[Title/Abstract])) OR (Arestin[Title/Abstract])) OR (Blemix[Title/Abstract])) OR (Cyclomin[Title/Abstract])) OR (Cyclops[Title/Abstract])) OR (Dentomycin[Title/Abstract])) OR (Dynacin[Title/Abstract])) OR (Icht-Oral[Title/Abstract])) OR (Icht Oral[Title/Abstract])) OR (Klinomycin[Title/Abstract])) OR (Lederderm[Title/Abstract])) OR (Mestacine[Title/Abstract])) OR (Minakne[Title/Abstract])) OR (Mino-Wolff[Title/Abstract])) OR (Mino Wolff[Title/Abstract])) OR (Minocin[Title/Abstract])) OR (Minocin MR[Title/Abstract])) OR (Minoclir[Title/Abstract])) OR (Minocycline	1,051	4:06:07

	Hydrochloride[Title/Abstract])) OR (Hydrochloride, Minocycline[Title/Abstract])) OR (Minocycline Monohydrochloride[Title/Abstract])) OR (Monohydrochloride, Minocycline[Title/Abstract])) OR (Minocycline, (4R-(4 alpha,4a beta,5a beta,12a beta))-Isomer[Title/Abstract])) OR (Minolis[Title/Abstract])) OR (Minomycin[Title/Abstract])) OR (Minoplus[Title/Abstract])) OR (Minotab[Title/Abstract])) OR (Akamin[Title/Abstract])) OR (Akne-Puren[Title/Abstract])) OR (Akne Puren[Title/Abstract])		
15	("Minocycline"[Mesh]) OR ((((((((((((((((((((((((((((((((((((((Minox 50[Title/Abstract]) OR (Aknemin[Title/Abstract])) OR (Aknin-Mino[Title/Abstract])) OR (Aknin Mino[Title/Abstract])) OR (Aknosan[Title/Abstract])) OR (Mynocine[Title/Abstract])) OR (Apo-Minocycline[Title/Abstract])) OR (Apo Minocycline[Title/Abstract])) OR (Arestin[Title/Abstract])) OR (Blemix[Title/Abstract])) OR (Cyclomin[Title/Abstract])) OR (Cyclops[Title/Abstract])) OR (Dentomycin[Title/Abstract])) OR (Dynacin[Title/Abstract])) OR (Icht-Oral[Title/Abstract])) OR (Icht Oral[Title/Abstract])) OR (Klinomycin[Title/Abstract])) OR (Lederderm[Title/Abstract])) OR (Mestacine[Title/Abstract])) OR (Minakne[Title/Abstract])) OR (Mino-Wolff[Title/Abstract])) OR (Mino Wolff[Title/Abstract])) OR (Minocin[Title/Abstract])) OR (Minocin MR[Title/Abstract])) OR (Minoclir[Title/Abstract])) OR (Minocycline Hydrochloride[Title/Abstract])) OR (Hydrochloride, Minocycline[Title/Abstract])) OR (Minocycline Monohydrochloride[Title/Abstract])) OR (Monohydrochloride, Minocycline[Title/Abstract])) OR (Minocycline, (4R-(4 alpha,4a beta,5a beta,12a beta))-Isomer[Title/Abstract])) OR (Minolis[Title/Abstract])) OR (Minomycin[Title/Abstract])) OR (Minoplus[Title/Abstract])) OR (Minotab[Title/Abstract])) OR (Akamin[Title/Abstract])) OR (Akne-Puren[Title/Abstract])) OR (Akne Puren[Title/Abstract]))	6,975	4:06:36
16	"Tetracycline"[Mesh]	20,582	4:07:28
17	(((((((((Tetrabid[Title/Abstract]) OR (4-Epitetracycline[Title/Abstract])) OR (4 Epitetracycline[Title/Abstract])) OR (Topicycline[Title/Abstract])) OR (Achromycin V[Title/Abstract])) OR (Hostacyclin[Title/Abstract])) OR (Tetracycline Hydrochloride[Title/Abstract])) OR (Tetracycline Monohydrochloride[Title/Abstract])) OR (Sustamycin[Title/Abstract])) OR (Achromycin[Title/Abstract])	1,104	4:09:09
18	("Tetracycline"[Mesh]) OR ((((((((((Tetrabid[Title/Abstract]) OR (4-Epitetracycline[Title/Abstract])) OR (4 Epitetracycline[Title/Abstract])) OR (Topicycline[Title/Abstract])) OR (Achromycin V[Title/Abstract])) OR (Hostacyclin[Title/Abstract])) OR (Tetracycline Hydrochloride[Title/Abstract])) OR (Tetracycline Monohydrochloride[Title/Abstract])) OR (Sustamycin[Title/Abstract])) OR (Achromycin[Title/Abstract]))	20,998	4:09:25

19	"Doxycycline"[Mesh]	10,307	4:10:19
20	(((((((((((((((((((((((((((((Doxycycline Monohydrate[Title/Abstract]) OR (Vibramycin[Title/Abstract])) OR (Atridox[Title/Abstract])) OR (Doxycycline Phosphate (1:1)[Title/Abstract])) OR (BMY-28689[Title/Abstract])) OR (BMY 28689[Title/Abstract])) OR (BMY28689[Title/Abstract])) OR (BU-3839T[Title/Abstract])) OR (BU 3839T[Title/Abstract])) OR (BU3839T[Title/Abstract])) OR (Doryx[Title/Abstract])) OR (Doxycycline Calcium Salt (1:2)[Title/Abstract])) OR (Doxycycline Hyclate[Title/Abstract])) OR (Doxycycline Hemiethanolate[Title/Abstract])) OR (Doxycycline Monohydrochloride, 6-epimer[Title/Abstract])) OR (Doxycycline Monohydrochloride, 6 epimer[Title/Abstract])) OR (Doxycycline Monohydrochloride, Dihydrate[Title/Abstract])) OR (Doxycycline Calcium[Title/Abstract])) OR (2-Naphthacenecarboxamide, 4-(dimethylamino)-1,4,4a,5,5a,6,11,12a-octahydro-3,5,10,12,12a-pentahydroxy-6-methyl-1,11-dioxo-, (4S-(4alpha,4aalpha,5alpha,5aalpha,6alpha,12aalpha))-[Title/Abstract])) OR (Alpha-6-Deoxyoxytetracycline[Title/Abstract])) OR (Alpha 6 Deoxyoxytetracycline[Title/Abstract])) OR (Doxycycline-Chinoin[Title/Abstract])) OR (Doxycycline Chinoin[Title/Abstract])) OR (Hydramycin[Title/Abstract])) OR (Oracea[Title/Abstract])) OR (Periostat[Title/Abstract])) OR (Vibra-Tabs[Title/Abstract])) OR (Vibra Tabs[Title/Abstract])) OR (Vibramycin Novum[Title/Abstract])) OR (Vibravenos[Title/Abstract])	676	4:15:28
21	("Doxycycline"[Mesh]) OR ((((((((((((((((((((((((((((((Doxycycline Monohydrate[Title/Abstract]) OR (Vibramycin[Title/Abstract])) OR (Atridox[Title/Abstract])) OR (Doxycycline Phosphate (1:1)[Title/Abstract])) OR (BMY-28689[Title/Abstract])) OR (BMY 28689[Title/Abstract])) OR (BMY28689[Title/Abstract])) OR (BU-3839T[Title/Abstract])) OR (BU 3839T[Title/Abstract])) OR (BU3839T[Title/Abstract])) OR (Doryx[Title/Abstract])) OR (Doxycycline Calcium Salt (1:2)[Title/Abstract])) OR (Doxycycline Hyclate[Title/Abstract])) OR (Doxycycline Hemiethanolate[Title/Abstract])) OR (Doxycycline Monohydrochloride, 6-epimer[Title/Abstract])) OR (Doxycycline Monohydrochloride, 6 epimer[Title/Abstract])) OR (Doxycycline Monohydrochloride, Dihydrate[Title/Abstract])) OR (Doxycycline Calcium[Title/Abstract])) OR (2-Naphthacenecarboxamide, 4-(dimethylamino)-1,4,4a,5,5a,6,11,12a-octahydro-3,5,10,12,12a-pentahydroxy-6-methyl-1,11-dioxo-, (4S-(4alpha,4aalpha,5alpha,5aalpha,6alpha,12aalpha))-[Title/Abstract])) OR (Alpha-6-Deoxyoxytetracycline[Title/Abstract])) OR (Alpha 6 Deoxyoxytetracycline[Title/Abstract])) OR (Doxycycline-Chinoin[Title/Abstract])) OR (Doxycycline	10,588	4:16:02

	Chinoin[Title/Abstract])) OR (Hydramycin[Title/Abstract])) OR (Oracea[Title/Abstract])) OR (Periostat[Title/Abstract])) OR (Vibra-Tabs[Title/Abstract])) OR (Vibra Tabs[Title/Abstract])) OR (Vibramycin Novum[Title/Abstract])) OR (Vibravenos[Title/Abstract]))		
22	((((((((((randomized controlled trial[Title/Abstract]) OR (controlled clinical trial[Title/Abstract])) OR (random allocation[Title/Abstract])) OR (double-blind[Title/Abstract])) OR (single-blind[Title/Abstract])) OR (survival[Title/Abstract])) OR (treatment[Title/Abstract])) OR (therapy[Title/Abstract])) OR (comparison[Title/Abstract])) OR (comparative[Title/Abstract])) OR (effective[Title/Abstract])) OR (efficacy[Title/Abstract])	8,722,636	4:18:59
23	((((((("Penicillins"[Mesh]) OR (((Antibiotics, Penicillin[Title/Abstract]) OR (Penicillin Antibiotics[Title/Abstract])) OR (Penicillin[Title/Abstract]))) OR (("Ceftriaxone"[Mesh]) OR (((((((((((((((((((((((((((((Ceftriaxon[Title/Abstract]) OR (Cefatriaxone[Title/Abstract])) OR (Ceftriaxone, Disodium Salt, Hemiheptahydrate[Title/Abstract])) OR (Ceftrex[Title/Abstract])) OR (Ceftriaxon Curamed[Title/Abstract])) OR (Ceftriaxon Hexal[Title/Abstract])) OR (Ceftriaxona Andreu[Title/Abstract])) OR (Ceftriaxona LDP Torlan[Title/Abstract])) OR (Ceftriaxone Irex[Title/Abstract])) OR (Ceftriaxone Sodium[Title/Abstract])) OR (Ceftriaxone Sodium, Anhydrous[Title/Abstract])) OR (Anhydrous Ceftriaxone Sodium[Title/Abstract])) OR (Lendacin[Title/Abstract])) OR (Longacef[Title/Abstract])) OR (Longaceph[Title/Abstract])) OR (Ro13-9904[Title/Abstract])) OR (Ro13 9904[Title/Abstract])) OR (Ro139904[Title/Abstract])) OR (Ro-13-9904[Title/Abstract])) OR (Ro 13-9904[Title/Abstract])) OR (Ro 13 9904[Title/Abstract])) OR (Ro 139904[Title/Abstract])) OR (Rocephin[Title/Abstract])) OR (Rocefalin[Title/Abstract])) OR (Rocephine[Title/Abstract])) OR (Rocefin[Title/Abstract])) OR (Tacex[Title/Abstract])) OR (Terbac[Title/Abstract])) OR (Ceftriaxone, Disodium Salt[Title/Abstract])) OR (Benaxona[Title/Abstract])) OR (Cefaxona[Title/Abstract])))) OR (("Erythromycin"[Mesh]) OR (((((((((((Erythromycin A[Title/Abstract]) OR (Erythromycin Phosphate[Title/Abstract])) OR (Phosphate, Erythromycin[Title/Abstract])) OR (Erythromycin Lactate[Title/Abstract])) OR (Lactate, Erythromycin[Title/Abstract])) OR (T-Stat[Title/Abstract])) OR (T Stat[Title/Abstract])) OR (TStat[Title/Abstract])) OR (Erymax[Title/Abstract])) OR (Erythromycin C[Title/Abstract])) OR (Erycette[Title/Abstract])) OR (Ilotycin[Title/Abstract])))) OR (("Minocycline"[Mesh]) OR ((((((((((((((((((((((((((((((((((((Minox 50[Title/Abstract]) OR (Aknemin[Title/Abstract])) OR (Aknin-Mino[Title/Abstract])) OR (Aknin Mino[Title/Abstract])) OR (Aknosan[Title/Abstract])) OR (Mynocine[Title/Abstract])) OR (Apo-Minocycline[Title/Abstract])) OR (Apo	3,431	4:23:33

	Minocycline[Title/Abstract])) OR (Arestin[Title/Abstract])) OR (Blemix[Title/Abstract])) OR (Cyclomin[Title/Abstract])) OR (Cyclops[Title/Abstract])) OR (Dentomycin[Title/Abstract])) OR (Dynacin[Title/Abstract])) OR (Icht-Oral[Title/Abstract])) OR (Icht Oral[Title/Abstract])) OR (Klinomycin[Title/Abstract])) OR (Lederderm[Title/Abstract])) OR (Mestacine[Title/Abstract])) OR (Minakne[Title/Abstract])) OR (Mino-Wolff[Title/Abstract])) OR (Mino Wolff[Title/Abstract])) OR (Minocin[Title/Abstract])) OR (Minocin MR[Title/Abstract])) OR (Minoclir[Title/Abstract])) OR (Minocycline Hydrochloride[Title/Abstract])) OR (Hydrochloride, Minocycline[Title/Abstract])) OR (Minocycline Monohydrochloride[Title/Abstract])) OR (Monohydrochloride, Minocycline[Title/Abstract])) OR (Minocycline, (4R-(4 alpha,4a beta,5a beta,12a beta))-Isomer[Title/Abstract])) OR (Minolis[Title/Abstract])) OR (Minomycin[Title/Abstract])) OR (Minoplus[Title/Abstract])) OR (Minotab[Title/Abstract])) OR (Akamin[Title/Abstract])) OR (Akne-Puren[Title/Abstract])) OR (Akne Puren[Title/Abstract])))) OR (("Tetracycline"[Mesh]) OR ((((((((((Tetrabid[Title/Abstract]) OR (4-Epitetracycline[Title/Abstract])) OR (4 Epitetracycline[Title/Abstract])) OR (Topicycline[Title/Abstract])) OR (Achromycin V[Title/Abstract])) OR (Hostacyclin[Title/Abstract])) OR (Tetracycline Hydrochloride[Title/Abstract])) OR (Tetracycline Monohydrochloride[Title/Abstract])) OR (Sustamycin[Title/Abstract])) OR (Achromycin[Title/Abstract])))) OR (("Doxycycline"[Mesh]) OR ((((((((((((((((((((((((((((((Doxycycline Monohydrate[Title/Abstract]) OR (Vibramycin[Title/Abstract])) OR (Atridox[Title/Abstract])) OR (Doxycycline Phosphate (1:1)[Title/Abstract])) OR (BMY-28689[Title/Abstract])) OR (BMY 28689[Title/Abstract])) OR (BMY28689[Title/Abstract])) OR (BU-3839T[Title/Abstract])) OR (BU 3839T[Title/Abstract])) OR (BU3839T[Title/Abstract])) OR (Doryx[Title/Abstract])) OR (Doxycycline Calcium Salt (1:2)[Title/Abstract])) OR (Doxycycline Hyclate[Title/Abstract])) OR (Doxycycline Hemiethanolate[Title/Abstract])) OR (Doxycycline Monohydrochloride, 6-epimer[Title/Abstract])) OR (Doxycycline Monohydrochloride, 6 epimer[Title/Abstract])) OR (Doxycycline Monohydrochloride, Dihydrate[Title/Abstract])) OR (Doxycycline Calcium[Title/Abstract])) OR (2-Naphthacenecarboxamide, 4-(dimethylamino)-1,4,4a,5,5a,6,11,12a-octahydro-3,5,10,12,12a-pentahydroxy-6-methyl-1,11-dioxo-, (4S-(4alpha,4aalpha,5alpha,5aalpha,6alpha,12aalpha))-[Title/Abstract])) OR (Alpha-6-Deoxyoxytetracycline[Title/Abstract])) OR (Alpha 6 Deoxyoxytetracycline[Title/Abstract])) OR (Doxycycline-Chinoin[Title/Abstract])) OR (Doxycycline		

	Chinoin[Title/Abstract])) OR (Hydramycin[Title/Abstract])) OR (Oracea[Title/Abstract])) OR (Periostat[Title/Abstract])) OR (Vibra-Tabs[Title/Abstract])) OR (Vibra Tabs[Title/Abstract])) OR (Vibramycin Novum[Title/Abstract])) OR (Vibravenos[Title/Abstract])))) AND (("Syphilis"[Mesh]) OR (Great Pox[Title/Abstract]))) AND (((((((((((randomized controlled trial[Title/Abstract]) OR (controlled clinical trial[Title/Abstract])) OR (random allocation[Title/Abstract])) OR (double-blind[Title/Abstract])) OR (single-blind[Title/Abstract])) OR (survival[Title/Abstract])) OR (treatment[Title/Abstract])) OR (therapy[Title/Abstract])) OR (comparison[Title/Abstract])) OR (comparative[Title/Abstract])) OR (effective[Title/Abstract])) OR (efficacy[Title/Abstract]))		

eTable 2 PubMed search strategy and result

No.	Query	Results
#10	#1 AND #8 AND [humans]/lim AND [clinical study]/lim	4149
#9	#1 AND #8	9273
#8	#2 OR #3 OR #4 OR #5 OR #6 OR #7	536834
#7	'doxycycline'/exp OR '4 (dimethylamino) 1, 4, 4a, 5, 5a, 6, 11, 12a octahydro 3, 5, 10, 12, 12a pentahydroxy 6 methyl 1, 11 dioxo 2 naphthacenecarboxamide' OR '5 hydroxy 6 deoxytetracycline' OR '6 deoxy 5 hydroxytetracycline' OR '6 deoxy 5 oxytetracycline' OR '6 deoxyoxytetracycline' OR '6 desoxy 5 hydroxytetracycline' OR '6beta deoxyoxytetracycline' OR 'adoxa' OR 'alpha 6 desoxy 5 oxytetracycline' OR 'amermycin' OR 'atrax' OR 'azudoxat' OR 'bactidox' OR 'banndoclin' OR 'basedillin' OR 'bassado' OR 'biocolyn' OR 'biodoxi' OR 'bronmycin' OR 'calcium doxycycline' OR 'cloran' OR 'cyclidox' OR 'dentistar' OR 'deoxycycline' OR 'deoxymycin dispersal' OR 'deoxymykoin' OR 'deoxyoxytetracycline' OR 'desoxy oxytetracycline' OR 'desoxycycline' OR 'doinmycin' OR 'dosil' OR 'dotur' OR 'doxaciclin' OR 'doxacycline' OR 'doxat' OR 'doxatet' OR 'doxi-sergo' OR 'doxibiotic' OR 'doxicycline' OR 'doxilin' OR 'doximed' OR	62776

	'doximycin' OR 'doxin' OR 'doxine' OR 'doxocycline' OR 'doxsig' OR 'doxy' OR 'doxy 100' OR 'doxy ii' OR 'doxy m' OR 'doxy n tablinen' OR 'doxy p ratiopharm' OR 'doxy puren' OR 'doxy s' OR 'doxy tablinen' OR 'doxy-1' OR 'doxy- caps' OR 'doxybiocin' OR 'doxycen' OR 'doxycen retard' OR 'doxychel' OR 'doxycin' OR 'doxycyclin' OR 'doxycycline' OR 'doxycycline calcium' OR 'doxycycline hydrate' OR 'doxycycline hydrochloride' OR 'doxycycline monohydrate' OR 'doxycycline ratiopharm' OR 'doxycydine monohydrate' OR 'doxylag' OR 'doxylin' OR 'doxymycin' OR 'doxypuren' OR 'doxytec' OR 'doxytrim' OR 'dumoxin' OR 'duracycline' OR 'esdoxin' OR 'etidoxina' OR 'gewacyclin' OR 'gs 3065' OR 'ibralene' OR 'idocyclin' OR 'idocyklin' OR 'interdoxin' OR 'investin' OR 'longamycin' OR 'lydox' OR 'magdrin' OR 'medomycin' OR 'mespafin' OR 'mildox' OR 'miraclin' OR 'monodox' OR 'nordox' OR 'nsc 56228' OR 'oracea' OR 'paldomycin' OR 'pernox gel' OR 'radox' OR 'remycin' OR 'respidox' OR 'roximycin' OR 'serodoxy' OR 'servidoxine' OR 'servidoxyne' OR 'siadocin' OR 'siclidon' OR 'sigadoxin' OR 'spanor' OR 'supracyclin' OR 'supramycina' OR 'tenutan' OR 'tolexine' OR 'tolexine ge' OR 'torymycin' OR 'tsurupioxin' OR 'unidox' OR 'veemycin' OR 'viadoxin' OR 'vibra s' OR 'vibra-s' OR 'vibrabiotic' OR 'vibracina' OR 'vibradox' OR 'vibramicina' OR 'vibramycin' OR 'vibramycin calcium' OR 'vibramycin monohydrate' OR 'vibramycin n' OR 'vibramycin-n' OR 'vibramycine' OR 'vibraveineuse' OR 'vibravenos' OR 'vibravenos sf' OR 'vibravet' OR 'viradoxyl-n' OR 'wanmycin' OR 'zadorin' OR 'zenavod'	
#6	'tetracycline'/exp OR 'achromycin' OR 'achromycin capsules' OR 'achromycin eye and ear ointment' OR 'achromycin for oral suspension' OR 'achromycin intravenous' OR 'achromycin ointment' OR 'achromycin ophthalmic oil suspension' OR 'achromycin ophthalmic sterilized' OR 'achromycin paediatric drops' OR 'achromycin pediatric drops' OR 'achromycin pharyngets' OR 'achromycin spersoids' OR 'achromycin surgical powder' OR 'achromycin syrup' OR 'achromycin tablets' OR 'achromycin troches' OR 'achromycin v' OR 'achromycin v capsules' OR 'achromycin v syrup' OR 'acromicina' OR 'acromycin' OR 'actisite' OR 'agromicina' OR 'akne pyodron' OR 'ala tet' OR 'ala-tet' OR 'ambamycin' OR 'ambramicina' OR 'ambramycin' OR 'ambrazoo' OR 'apo tetra' OR 'apo-tetra' OR 'apocyclin' OR 'apotetra' OR 'artomycin' OR 'austramycin' OR 'beatacycline' OR 'bicycline' OR 'bio tetra' OR 'biotetra' OR 'bitacycline' OR 'bristaciclina' OR 'bristacyclin' OR 'bristacyclina' OR 'bristacycline' OR 'bristacycline intravenous' OR 'bristocycline' OR 'brodspec' OR 'cadicycline' OR 'calociclina' OR 'cefracycline' OR 'ciclotetryl' OR 'clinitetrin' OR 'clinitetrin syrup' OR 'combicyclin' OR 'conmycin' OR 'copharlan' OR 'criseociclina' OR 'cyclabid' OR 'cyclindif' OR 'cyclomycetin' OR 'cyclomycin' OR 'cyclomycine' OR 'cyclopar' OR 'cyclopen' OR 'deschloraureomycin' OR 'deschlorbiomycin' OR 'deschloroaureomycin' OR 'deschlorobiomycin' OR 'dhatracin' OR 'diaciclin' OR 'dicyclin forte' OR 'dumocyclin' OR 'dumocyclina' OR 'dumocycline' OR 'economycin' OR 'enkacyclin' OR 'epsilontetracycline' OR 'fabacyclin' OR 'farciclina' OR 'fermentmycin' OR 'fermycine' OR 'floramicina' OR 'florocycline' OR 'hestacyclin' OR 'hostaciclina' OR 'hostacyclin' OR 'hostacycline' OR 'hostacycline p' OR 'hostacycline-p' OR 'hydracycline' OR 'hydromycin' OR 'ibicyn' OR 'ikacycline' OR 'italcycline' OR 'kemoclin' OR 'kristacyclin' OR 'latycin' OR 'lenocin' OR 'mediacycline' OR 'medicyclin' OR 'medocycline' OR 'mervacycline' OR 'micipan' OR 'murazine' OR 'mysteclin' OR 'mystecline' OR	112776

	'myszeklin' OR 'neocycline' OR 'neotetrine' OR 'novotetra' OR 'ofticlin' OR 'omegamycin' OR 'omnaze' OR 'orencyclin f 500' OR 'orencyclin f- 500' OR 'oricyclin' OR 'panciclina' OR 'pancycline wirkstoff' OR 'panmycin' OR 'panmycin hydrochloride' OR 'panmycin p' OR 'panmycin syrup' OR 'pansan' OR 'pantocycline' OR 'parenciclina' OR 'pervasol' OR 'polarcyclin' OR 'polfamycin' OR 'polycycline' OR 'polycycline hydrochloride' OR 'polycycline suspension' OR 'polycyline' OR 'polyotic' OR 'porcycline' OR 'premocycline' OR 'purocyclina' OR 'quadricycline' OR 'quimocyclar' OR 'quirvetin' OR 'recycline' OR 'remicyclin' OR 'reseomycin' OR 'resomicina' OR 'resteclin' OR 'retet' OR 'ricycline' OR 'rimatet' OR 'robicyclan' OR 'robitet' OR 'sanclomycine' OR 'sarocycline' OR 'servitet' OR 'spaciclina' OR 'steclin' OR 'steclin v' OR 'stilciclina' OR 'subamycin' OR 'supramycin' OR 'tefilin' OR 'teracyn' OR 'tesyklin' OR 'tetra atlantis' OR 'tetra central' OR 'tetra wolf' OR 'tetra-atlantis' OR 'tetrabakat' OR 'tetrabid' OR 'tetrabien' OR 'tetrabioptal' OR 'tetrabior' OR 'tetrablet' OR 'tetrabon' OR 'tetrachel' OR 'tetraciclene' OR 'tetraciclina' OR 'tetraciklin' OR 'tetracitro' OR 'tetracitro s' OR 'tetracon' OR 'tetracyclin' OR 'tetracycline' OR 'tetracycline hcl' OR 'tetracycline hydrochloride' OR 'tetracycline solution' OR 'tetracycline syrup buffered' OR 'tetracyline' OR 'tetracyn' OR 'tetracyn capsules' OR 'tetracyn intravenous' OR 'tetracyn paediatric drops' OR 'tetracyn pediatric drops' OR 'tetracyn syrup' OR 'tetracyn tablets' OR 'tetradecin' OR 'tetrafil' OR 'tetralen' OR 'tetralonga' OR 'tetralution' OR 'tetramax' OR 'tetramed' OR 'tetramig' OR 'tetrana' OR 'tetranase' OR 'tetrano' OR 'tetraplus' OR 'tetrarco' OR 'tetrarco l.a.' OR 'tetraseptin' OR 'tetrasuiss' OR 'tetrecu' OR 'tetrex paediatric drops' OR 'tetrex pediatric drops' OR 'tetrex syrup' OR 'tetrosol' OR 'thuricyclin' OR 'topicycline' OR 'totomycin' OR 'triclina' OR 'triphacyclin' OR 'tsiklomitsin' OR 'umetracil' OR 'veracin'	
#5	'minocycline'/exp OR '4, 7 bis (dimethylamino) 1, 10, 11, 12a tetrahydroxy 3, 12 dioxo 4a, 5, 5a, 6 tetrahydro 4h tetracene 2 carboxamide' OR '4, 7 bis (dimethylamino) 1, 4, 4a, 5, 5a, 6, 11, 12a octahydro 3, 10, 12, 12a tetrahydroxy 1, 11 dioxo 2 naphthacenecarboxamide' OR '7 dimethylamino 6 demethyl 6 deoxytetracycline' OR 'akamin' OR 'aknemin' OR 'aknosan' OR 'amzeeq' OR 'arestin' OR 'borymycin' OR 'cyclimycin' OR 'cynomycin' OR 'dynacin' OR 'klinomycin' OR 'klinotab' OR 'lederderm' OR 'logryx' OR 'menocycline' OR 'mestacine' OR 'micromycin' OR 'minaxen' OR 'mino-50' OR 'mino-wolff' OR 'minocin' OR 'minocin g' OR 'minocin mr' OR 'minocin pf' OR 'minoclin' OR 'minoclir 50' OR 'minocyclin' OR 'minocyclin 50 stada' OR 'minocycline' OR 'minocycline hydrochloride' OR 'minocyn' OR 'minogalen' OR 'minoline' OR 'minolira' OR 'minomax' OR 'minomycin' OR 'minotab' OR 'minotab 50' OR 'minotab-100' OR 'mirosin' OR 'mynocine' OR 'romin' OR 'skinocyclin' OR 'solodyn' OR 'spicline' OR 'vectran' OR 'vectrin' OR 'ximino' OR 'zilxi'	27372
#4	'erythromycin'/exp OR 'a/t/s' OR 'abomacetin' OR 'acneryne' OR 'acnesol' OR 'akne mycin' OR 'akne-mycin' OR 'aknederm ery gel' OR 'aknemycin' OR 'anamycin' OR 'bonac gel' OR 'c-solve-2' OR 'cliniderm' OR 'deripil' OR 'duraerythromycin' OR 'e mycin' OR 'e-base' OR 'e-glades' OR 'e-mycin' OR 'e-solve 2' OR 'emgel' OR 'emu v' OR 'emu-v' OR 'emu-ve' OR 'emuvin' OR 'emycin'	

	OR 'eriecu' OR 'erimycin-t' OR 'eriprodin' OR 'eritimix' OR 'eritrex' OR 'eritrocina' OR 'eritromicina' OR 'erixyl' OR 'ermycin' OR 'ermysin' OR 'ery maxin' OR 'ery-b' OR 'ery-diolan' OR 'ery-maxin' OR 'ery-tab' OR 'eryacne' OR 'eryacnen' OR 'eryc' OR 'eryc 125' OR 'eryc ld' OR 'eryc sprinkles' OR 'eryc-125' OR 'eryc-250' OR 'erycen' OR 'erycette' OR 'erycin' OR 'erycinum' OR 'eryderm' OR 'erydermec' OR 'erydermer' OR 'eryfluid' OR 'erygel' OR 'eryhexal' OR 'erymax' OR 'erymaxin' OR 'erymed' OR 'erysafe' OR 'erystrat' OR 'erytab' OR 'eryth mycin' OR 'eryth-mycin' OR 'erythelan' OR 'erythmycin' OR 'erythomycin' OR 'erythra-derm' OR 'erythran' OR 'erythro 200' OR 'erythro teva' OR 'erythro-statin' OR 'erythro-teva' OR 'erythrogan' OR 'erythrogel' OR 'erythrogran' OR 'erythroguent' OR 'erythromid' OR 'erythromycin' OR 'erythromycin a' OR 'erythromycin a dihydrate' OR 'erythromycin base' OR 'erythromycin base filmtab' OR 'erythromycin delayed release capsules' OR 'erythromycine' OR 'erythromycinum' OR 'erythroteva' OR 'erytop' OR 'erytrarco' OR 'erytrociclin' OR 'etinycine' OR 'etrolate' OR 'etromycin' OR 'ilocap' OR 'ilocaps' OR 'iloticina' OR 'ilotycin' OR 'ilotycin t.s.' OR 'inderm gel' OR 'labocne' OR 'latotryd' OR 'lederpax' OR 'mephamycin' OR 'oftalmolosa cusi eritromicina' OR 'oftalmolosa cusi erythromycin' OR 'oftamolets' OR 'pantodrin' OR 'pantomycin' OR 'pantomycin forte' OR 'pantomycin s 400' OR 'pce' OR 'pce dispertab' OR 'pce dispertabs' OR 'pharyngocin' OR 'primacine' OR 'r-p mycin' OR 'robimycin' OR 'romycin' OR 'roymicin' OR 'rp mycin' OR 'rythocin' OR 'sans-acne' OR 'sansac' OR 'skid gel e' OR 'staticin' OR 'stiemycin' OR 'stimycine' OR 't stat' OR 't-stat' OR 'theramycin' OR 'theramycin z'	95148
#3	'ceftriaxone'/exp OR '7 [2 (2 aminothiazol 4 yl) glyoxylamido] 3 [[(2, 5 dihydro 6 hydroxy 2 methyl 5 oxo 1, 2, 4 triazin 3 yl) thio] methyl] 2 cephem 2 carboxylic acid 7 2 (o methyloxime)' OR 'acantex' OR 'axone' OR 'benaxona' OR 'biotrakson' OR 'biotriax' OR 'bioxon' OR 'broadced' OR 'brospec' OR 'cef-3' OR 'cefaflox' OR 'cefalogen' OR 'cefatriaxone' OR 'cefaxona' OR 'cefaxone' OR 'cefin' OR 'cefotal' OR 'cefotriaxon' OR 'cefotriaxone' OR 'cefriex' OR 'ceftrex' OR 'ceftrian' OR 'ceftriaxone' OR 'ceftriaxone and dextrose in duplex container' OR 'ceftriaxone sodium' OR 'ceftrilem' OR 'cefxon' OR 'ceph 3 em 4 carboxylic acid 7 2 (o methyloxime), 7 [2 (2 aminothiazol 4 yl) glyoxylamido] 3 [[(2, 5 dihydro 6 hydroxy 2 methyl 5 oxo 1, 2, 4 triazin 3yl) thio] methyl]' OR 'cephin' OR 'cephtriaxone' OR 'cerixon' OR 'cikedrix' OR 'citeral (ceftriaxone)' OR 'ecotrixon' OR 'elpicef' OR 'eurocef' OR 'exempla (drug)' OR 'ferfacef' OR 'forgram' OR 'glicocef' OR 'gomcephin' OR 'grifotriaxona' OR 'incephin' OR 'keftriaxon' OR 'keptrix' OR 'lopratin' OR 'lyceft' OR 'medoxonum' OR 'megion' OR 'mesporin' OR 'mesporin im' OR 'mesporin iv' OR 'monocef' OR 'nakaxone' OR 'novosef' OR 'oframax' OR 'pantrixon' OR 'retrokor' OR 'rinxofay' OR 'ro 13 9904' OR 'rocefalin roche' OR 'rocefin' OR 'rocephalin' OR 'rocephin' OR 'rocephin biochemie' OR 'rocephin im' OR 'rocephin im convenience kit' OR 'rocephin iv' OR 'rocephin roche' OR 'rocephin with dextrose' OR 'rocephine' OR 'rocephine im' OR 'rocephine iv' OR 'rocephine roche' OR 'rocidar' OR 'rowecef' OR 'roxcef' OR 'roxon' OR 'samixon' OR 'sintrex' OR 'socef' OR 'sunflow' OR 'tacex' OR 'torocef-1' OR 'trexofin' OR 'triaken' OR 'triax' OR 'triaxone' OR 'tricefin' OR 'tricephin' OR 'trijec' OR 'xtenda' OR 'zefaxone' OR 'zefone 250'	71522

#2	'penicillin derivative'/exp OR 'antibiotics, penicillins' OR 'penicillin' OR 'penicillin derivate' OR 'penicillin derivative' OR 'penicillin series' OR 'penicillins' OR 'penicillium extract'	362538
#1	'syphilis'/exp OR 'early syphilis':ti,ab,kw OR 'lues':ti,ab,kw OR 'syphilis':ti,ab,kw OR 'syphilitic disorder':ti,ab,kw 45854 OR 'venereal syphilis':ti,ab,kw	45854

eTable 3 Embase search strategy and result

Study	Selection of research subjects				Comparability		Outcome			Quality score
	1	2	3	4	5	6	7	8	9	-
Thomas Bettuzzi 2021	1	0	1	1	1	1	1	0	1	7
Haoqing Wu 2021	1	1	1	1	1	1	1	1	1	9
Marilia B. Antonio 2019	1	1	1	1	1	1	1	0	1	8
Li-Li Shao 2016	1	1	1	1	1	0	1	1	1	8
Jen-Chih Tsai 2014	1	0	1	1	1	1	1	0	1	7
K.C. Psomas 2012	1	0	1	1	1	0	1	1	1	7
P.Spornraft-Ragaller 2011	1	1	1	1	1	0	1	0	1	7
Khalil G. Ghanem 2006	1	0	1	1	1	1	1	0	1	7
Mark E.Dowell 1992	1	0	1	1	1	0	1	0	1	6
Nicholas J.Fiumara 1978	1	0	1	1	1	0	1	1	1	7
Nicholas J.Fiumara 1977	1	0	1	1	1	0	1	1	1	7
Nicholas J.Fiumara 1977	1	0	1	1	1	0	1	1	1	7
Arnold L.Schroeter 1972	1	0	1	1	1	0	1	1	1	7
JAmes B. LUCAS 1967	1	0	1	1	1	0	1	1	1	7

Selection of research subjects:

1. Representation of the included population. 2. Penicillin group and alternative group from the same source population?3. Medication administration described in detail. 4.Demonstration that outcome of interest was not present at start of study.

Comparability: 5.Control of important confounding factors during study design.6.Statistical methods were used to control for significant confounders during analysis

Outcome: 7. ≥2 years of followed-up time . 8. Clear definition of serological response. 9.Adequacy of follow up of cohorts.

eTable 4 Results of quality assessment using Newcastle

eTable 5-1 Results of the head to head meta-analysis on serological response at 3-month follow-up

Comparison of interventions	No. of studies	RR (95%CI)	Heterogeneity P value	I2(%)
Ceftriaxone vs. Penicillin	3	1.05 [0.92; 1.20]	0.70	0
Tetracycline vs. Penicillin	3	1.01 [0.99; 1.04]	0.37	0
Erythromycin vs. Penicillin	2	0.98 [0.95; 1.00]	0.96	0
Erythromycin vs. Tetracycline	2	1.03 [1.00; 1.06]	0.6	0

eTable 5-2 Results of the head-to-head meta-analysis on serological response at 6-month follow-up

Comparison of interventions	No. of studies	RR (95%CI)	Heterogeneity P value	I2(%)
Ceftriaxone vs. Penicillin	3	**1.13 [1.03; 1.25]**	0.51	0
Tetracycline vs. Penicillin	6	0.98 [0.93; 1.03]	0.58	0
Doxycycline vs. Penicillin	1	0.88 [0.75; 1.02]	-	-
Erythromycin vs. Penicillin	2	0.91 [0.80; 1.03]	0.2	39
Erythromycin vs. Tetracycline	2	0.91 [0.78; 1.06]	0.16	49

eTable 5-3 Results of the head to head meta-analysis on serological response at 12-month follow-up

Comparison of interventions	No. of studies	RR (95%CI)	Heterogeneity P value	I^2(%)
Ceftriaxone vs. Penicillin	5	1.03 [0.91; 1.17]	0.31	17
Tetracycline vs. Penicillin	3	1.04 [0.87; 1.25]	0.12	52
Doxycycline vs. Penicillin	4	1.04 [0.98; 1.10]	0.65	0
Erythromycin vs. Penicillin	2	0.64 [0.28; 1.46]	0.08	66
Erythromycin vs. Tetracycline	2	0.61 [0.22; 1.69]	0.04	76
Ceftriaxone vs. Doxycycline	1	1.06 [0.75; 1.49]	-	-

eTable 5-4 Results of the head to head meta-analysis on serological response at 24-month follow-up

Comparison of interventions	No. of studies	RR (95%CI)	Heterogeneity P value	I^2(%)
Minocycline vs. Penicillin	2	0.99 [0.90; 1.07]	0.54	0
Tetracycline vs. Penicillin	3	1.00 [0.96; 1.03]	0.7	0
Erythromycin vs. Penicillin	1	0.83 [0.67; 1.02]	-	-
Erythromycin vs. Tetracycline	1	0.86 [0.68; 1.09]	-	-

eTable 5 Direct meta-analysis for different follow-up time

eTable 6-1 Data on serological response rate of interventions at 3-month follow-up

Study	Intervention	Response	Total patients	Response rate
Yuping Cao 2017	Penicillin	86	115	75%
	Ceftriaxone	86	110	78%
P. Spornraft-Ragaller 2011	Penicillin	5	7	71%
	Ceftriaxone	10	11	91%
H.Schofer 1989	Penicillin	8	10	80%
	Ceftriaxone	10	13	77%
Nicholas J.Fiumara 1977	Penicillin	83	175	47%
	Tetracycline	8	21	38%
Arnold L.Schroeter 1972	Penicillin	230	233	99%
	Tetracycline	98	99	99%
	Erythromycin	180	187	96%
James B.Lucas 1967	Penicillin	116	119	97%
	Erythromycin	40	42	95%
	Tetracycline	46	46	100%

eTable 6-2 Data on serological response rate of interventions at 6-month follow-up

Study	Intervention	Response	Total patients	Response rate
Thomas Bettuzzi 2021	Penicillin	76	93	82%
	Ceftriaxone	21	24	88%
Yuping Cao 2017	Penicillin	92	118	78%
	Ceftriaxone	101	112	90%
Jen-Chih Tsai 2014	Penicillin	196	271	72%
	Doxycycline	78	123	63%
Mark E.Dowell 1992	Penicillin	8	13	62%
	Ceftriaxone	28	43	65%
H.Schofer 1989	Penicillin	2	2	100%
	Ceftriaxone	4	6	67%
T.Thiru Moorthy 1987	Penicillin	1	5	20%
	Ceftriaxone	5	13	38%
Nicholas J.Fiumara 1978	Penicillin	140	252	56%
	Tetracycline	11	23	48%

Nicholas J.Fiumara 1977	Penicillin	139	175	79%
	Tetracycline	14	21	67%
Arnold L.Schroeter 1972	Penicillin	190	198	96%
	Tetracycline	82	87	94%
	Erythromycin	138	153	90%
James B.Lucas 1967	Penicillin	76	82	93%
	Erythromycin	21	29	72%
	Tetracycline	28	30	93%

eTable 6-3 Data on serological response rate of interventions at 12-month follow-up

Study	Intervention	Response	Total patients	Response rate
Marilia B. Antonio 2019	Penicillin	83	115	72%
	Doxycycline	35	50	70%
Yuping Cao 2017	Penicillin	96	118	81%
	Ceftriaxone	103	112	92%
Jen-Chih Tsai 2014	Penicillin	185	271	68%
	Doxycycline	60	91	66%
K.C. Psomas 2012	Penicillin	39	52	75%
	Doxycycline	11	15	73%
	Ceftriaxone	38	49	78%
P.Spornraft-Ragaller 2011	Penicillin	11	11	100%
	Ceftriaxone	11	12	92%
Khalil G. Ghanem 2006	Penicillin	69	73	95%
	Doxycycline	34	34	100%
H.Schofer 1989	Penicillin	5	7	71%
	Ceftriaxone	3	5	60%
T.Thiru Moorthy 1987	Penicillin	2	4	50%
	Ceftriaxone	6	13	46%
Nicholas J.Fiumara 1978	Penicillin	164	252	65%
	Tetracycline	18	23	78%
Arnold L.Schroeter 1972	Penicillin	143	150	95%
	Tetracycline	60	67	90%
	Erythromycin	87	106	82%
James B.Lucas 1967	Penicillin	15	21	71%
	Erythromycin	3	12	25%

	Tetracycline	8	10	80%

eTable 6-4 Data on serological response rate of interventions at 24-month follow-up

Study	Intervention	Response	Total patients	Response rate
Haoqing Wu 2021	Penicillin	104	118	88%
	Minocycline	135	158	85%
Li-Li Shao 2016	Penicillin	31	40	78%
	Minocycline	125	156	80%
Nicholas J.Fiumara 1978	Penicillin	234	252	93%
	Tetracycline	20	23	87%
Nicholas J.Fiumara 1977	Penicillin	165	165	100%
	Tetracycline	39	39	100%
Arnold L.Schroeter 1972	Penicillin	60	68	88%
	Tetracycline	34	40	85%
	Erythromycin	27	37	73%

eTable 6 Serological response rate data of intervention measures in different follow-up time

Outcome index	Loop	IF&95%CI	P
Serological response rates at the 6-month follow-up	penicillin-tetracycline-erythromycin	0.109(0.00,0.33)	0.342
Serological response rates at the 12-month follow-up	penicillin-tetracycline-erythromycin	0.24(0.00,0.52)	0.092
Serological response rates at the 12-month follow-up	penicillin-doxycycline-ceftriaxone	0(0.00,0.36)	1

eTable 7 Loop inconsistency test results

Ceftriaxone	1.05 (0.91,1.21)	1.05 (0.86,1.29)	1.22 (1.01,1.48)	1.06 (0.94,1.20)
1. 28(1. 07, 1. 53)	Doxycycline	1.01 (0.84,1.21)	1.17 (0.98,1.40)	1.01 (0.91,1.12)
1. 15(1. 04, 1. 28)	0. 90(0. 77, 1. 06)	Tetracycline	1.16 (0.98,1.37)	1.01 (0.87,1.16)
1. 21(1. 09, 1. 35)	0. 95(0. 80, 1. 11)	1. 05(0. 98, 1. 12)	Erythromycin	0.87 (0.75,1.00)
1. 12(1. 02, 1. 23)	0. 88(0. 75, 1. 02)	0. 97(0. 93, 1. 02)	0. 93(0. 88, 0. 98)	Penicillin

Paired comparison of serological reaction rates of 5 antibiotics at 6-month follow-up (lower left corner)and 12-month follow-up(top right corner). Data are RRs and 95% CIs in each grid. If both the RR and 95% CI are higher than 1 it indicates that the column-defining antibiotic's efficacy is better than that of row-defining antibiotic.

eTable 8 Serological response rates league table at the 6-month follow-up and 12-month follow-up

Reference	Dosage and duration
Bettuzzi 2021	BenPen 3-4 MU i.v. every 4 h
	Ceftriaxone 2 g i.v. once daily for 10 days
Dowell 1992	BenPen three doses each of 2.4 MU at weekly intervals
	Ceftriaxone 1 to 2 g daily for 10 to 14 days
Cao 2017	BenPen 2.4 MU i.m. weekly for 2 weeks
	Ceftriaxone 1 g i.v. once daily for 10 days
Schofer 1989	BenPen 1 MIU i.m. daily for 15 days
	Ceftriaxone 4×1 g i.m. every 2 days
Moorthy 1987	BenPen 2.4 MU a single i.m.
	Ceftriaxone 3 g in a single i.m. or 2 g i.m. daily for 2 days or 2 g i.m. daily for 5 days

Notes: BenPen: benzathine penicillin G; i.m.: intramuscular injection; i.v.: intravenously injection.

eTable 9 Treatment options for neurosyphilis

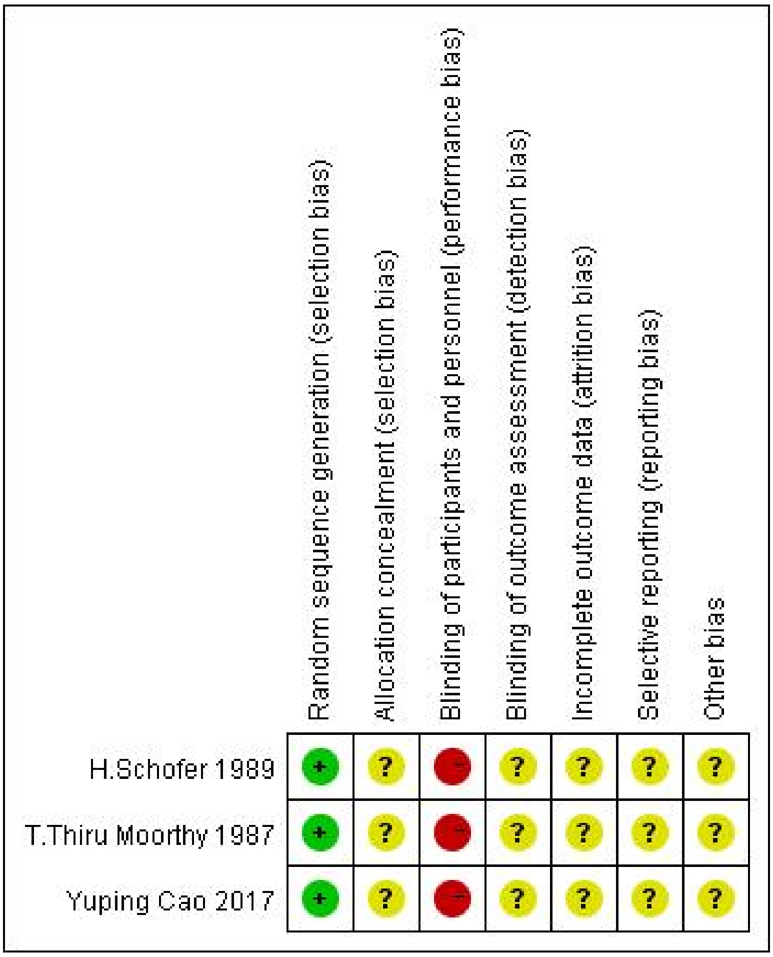

eFigure 1 Risk of bias summary

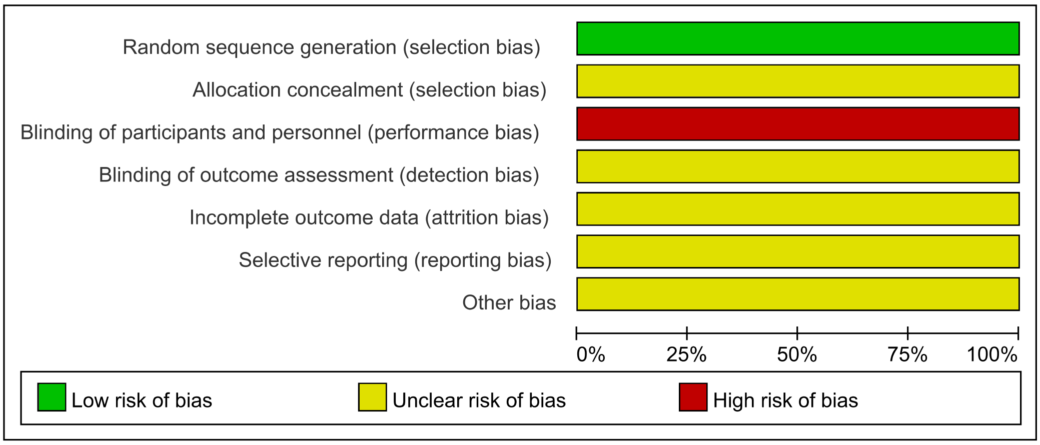

eFigure 2 Risk of bias graph

A

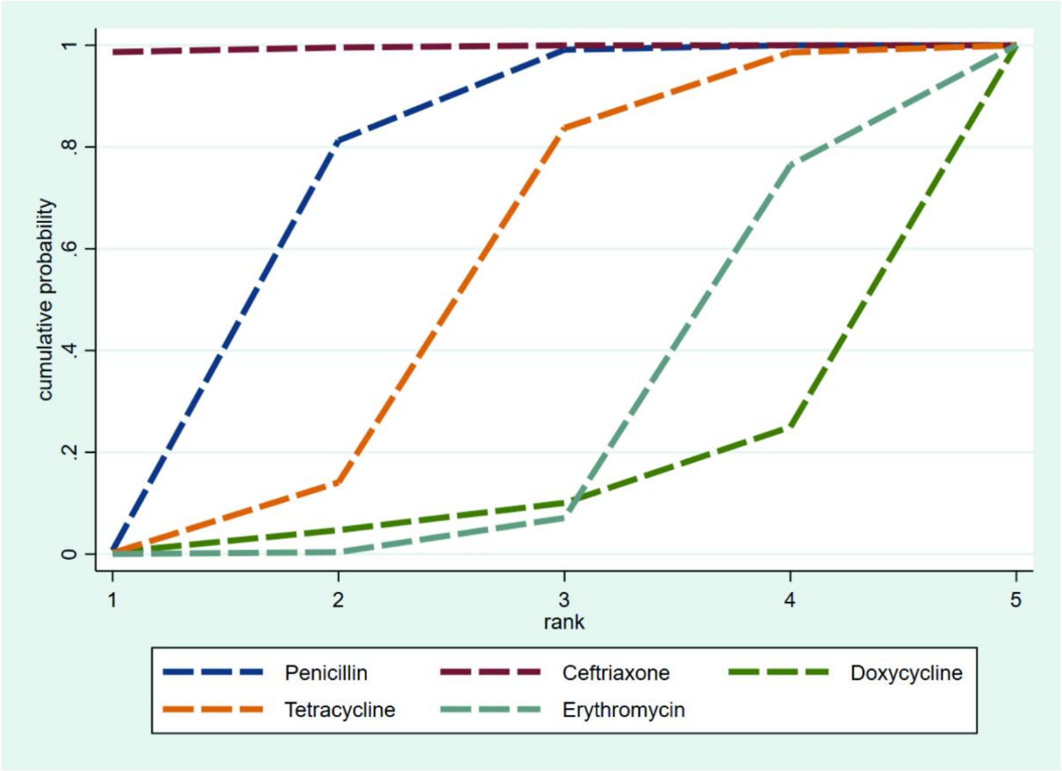

B

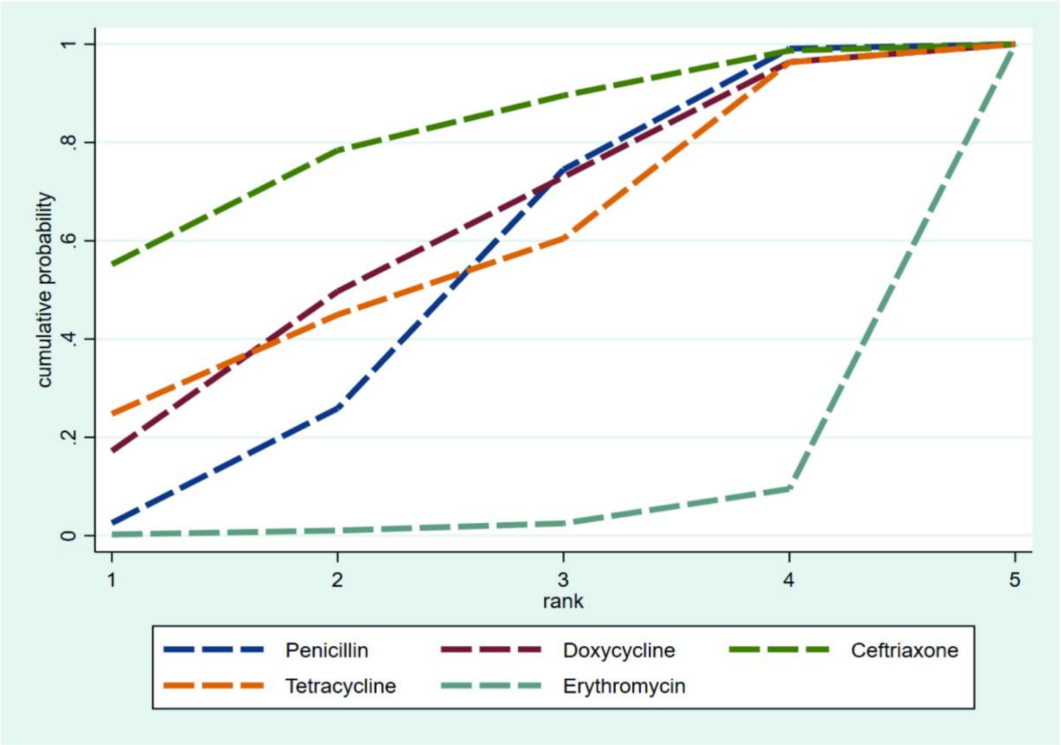

eFigure 3 Cumulative ranking probability curves of serological response rates at 6-month follow-up (A) and 12-month follow-up (B)

A

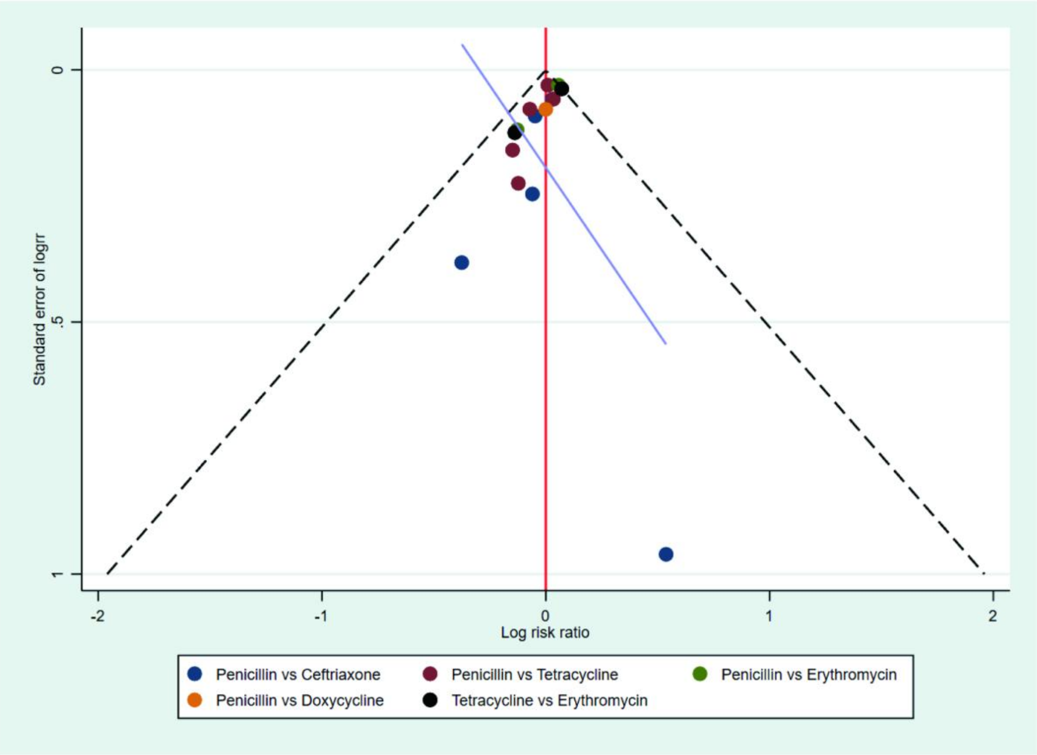

B

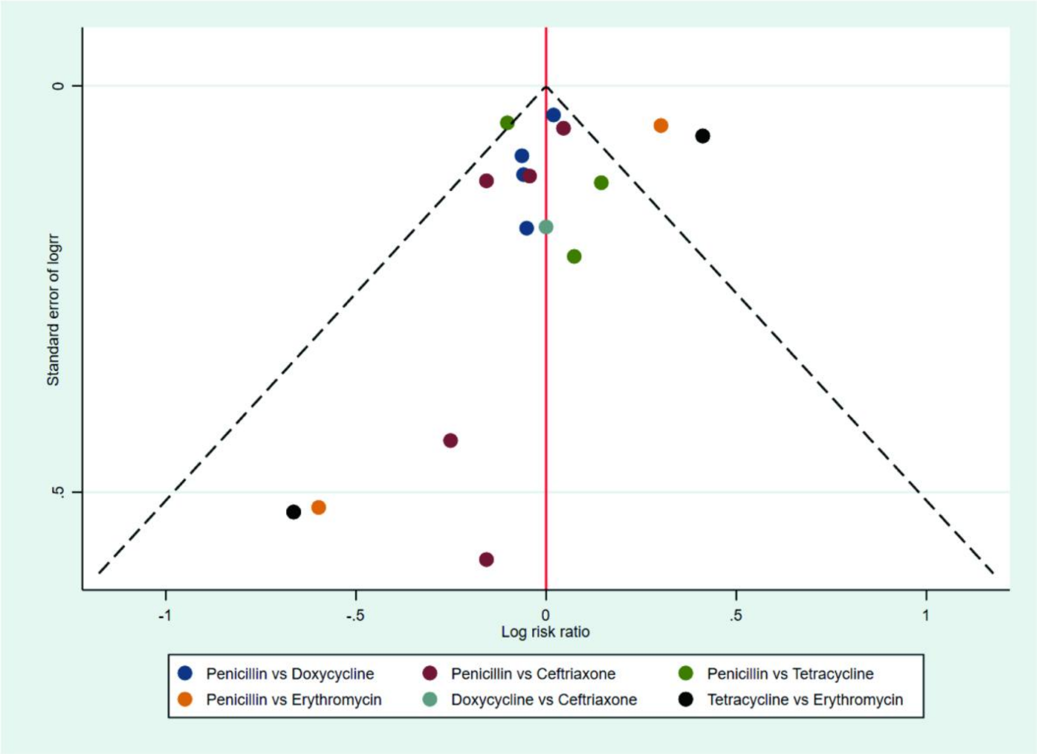

eFigure 4 Funnel plots of serological response rates at the 6-month follow-up (A) and 12-month follow-up (B)

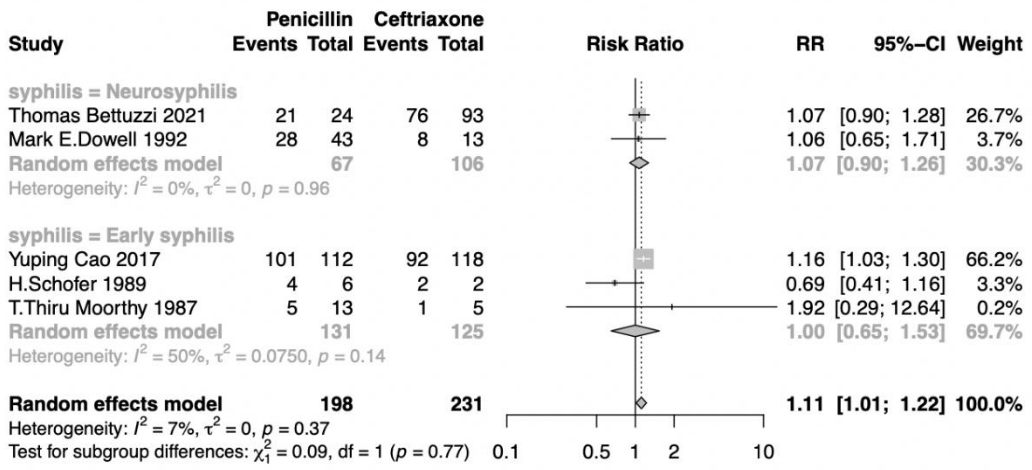

eFigure 5 Subgroup analysis of ceftriaxone versus penicillin for treatment of neurosyphilis and early syphilis at the 6-month follow-up
